# Extent of threats to marine fish from the online aquarium trade in the United States

**DOI:** 10.1111/cobi.70155

**Published:** 2025-10-08

**Authors:** Bing Lin, Yiwen Zeng, Bryan To, Robert J. Holmberg, Andrew L. Rhyne, Michael Tlusty, David S. Wilcove

**Affiliations:** ^1^ School of Public and International Affairs Princeton University Princeton New Jersey USA; ^2^ Thriving Oceans Research Hub University of Sydney Sydney New South Wales Australia; ^3^ The Asian School of the Environment Nanyang Technological University Singapore Singapore; ^4^ Department of Ecology and Evolutionary Biology Princeton University Princeton New Jersey USA; ^5^ Department of Biology, Marine Biology and Environmental Science Roger Williams University Bristol Rhode Island USA; ^6^ Center for Economic and Environmental Development Roger Williams University Bristol Rhode Island USA; ^7^ School for the Environment University of Massachusetts Boston Boston Massachusetts USA; ^8^ Global Ocean Conservation Program Monterey Bay Aquarium Monterey California USA

**Keywords:** aquaculture, captive breeding, e‐commerce, marine aquarium trade, marine conservation, online, ornamental fish, saltwater fish, wild capture, wildlife trade, acuacultura, captura silvestre, comercio electrónico, conservación marina, crianza en cautiverio, en línea, mercado de acuarios marinos, mercado de fauna, peces marinos, peces ornamentales, 海洋水族馆贸易, 水产养殖, 观赏鱼, 海洋保护, 人工繁殖, 野生捕捞, 海水鱼, 在线, 电子商务, 野生动物贸易

## Abstract

The global marine aquarium hobby is a multibillion‐dollar industry, largely driven by demand from the United States. Much of this trade occurs online. We web scraped 4 major US‐based e‐commerce platforms selling marine aquarium fish to determine the retail price and source (wild capture, aquaculture, or both) of 13 families of ray‐finned marine fish (*Actinopterygii*). We supplemented this with ecological and economic trait data from FishBase and the International Union for Conservation of Nature (IUCN). Across all platforms and 13 popular fish taxonomic families, we found 734 unique species for sale, 89.2% (655 species) of which were sourced exclusively from the wild. A total of 45 species were of conservation concern (20 threatened species and 25 additional species with decreasing population trends), 38 of which were sourced solely from the wild. Retail price was significantly correlated with source, body length, minimum occupied depth, and schooling behavior. A further 100 species for sale were not listed as being in the aquarium trade in FishBase or by the IUCN, indicating incomplete information on this fishery in 2 important databases. For 58 species (encompassing 71 variants) with both wild‐caught and captive‐bred individuals for sale, aquaculture fish were a mean 28.1% (95% confidence interval 15.3) cheaper than their wild‐caught counterparts.

## Abstract (es)

Extensión de las amenazas a los peces marinos por el mercado de acuarios en los Estados Unidos

**Resumen:**

La afición mundial por los acuarios marinos es una industria multimillonaria, impulsada en gran medida por la demanda de Estados Unidos. Gran parte de este comercio se realiza en línea. Recopilamos datos de cuatro importantes plataformas de comercio electrónico con sede en Estados Unidos que venden peces para acuarios marinos con el fin de determinar el precio de venta al público y el origen (captura silvestre, acuicultura o ambos) de 13 familias de peces marinos con aletas radiadas (Actinopterygii). Complementamos esta información con datos sobre características ecológicas y económicas de FishBase y la Unión Internacional para la Conservación de la Naturaleza (UICN). En todas las plataformas y las 13 familias taxonómicas de peces más populares, encontramos 734 especies únicas a la venta, de las cuales el 89.2% (655 especies) procedían exclusivamente de la naturaleza. Cuarenta y cinco especies eran objeto de interés por su conservación (20 especies amenazadas y 25 especies adicionales con tendencias de disminución de la población), 38 de las cuales procedían exclusivamente de la naturaleza. El precio de venta al público estaba significativamente correlacionado con el origen, la longitud corporal, la profundidad mínima ocupada y el comportamiento gregario. Además, 100 especies a la venta no figuraban como comercializadas en acuarios en FishBase ni en la UICN, lo que indica que la información sobre esta pesquería es incompleta en dos importantes bases de datos. En el caso de 58 especies (que abarcan 71 variantes) con ejemplares capturados en estado silvestre y criados en cautividad a la venta, los peces de acuicultura eran, en promedio, un 28.1 % (IC del 95%: 15.3) más baratos que sus homólogos capturados en estado silvestre.

## 通过社会‐生态指标集合在基于区域的保护监测中考虑人与自然的联系

**【摘要】** 全球海洋水族爱好是一个数十亿美元的产业, 主要由美国的需求所驱动。这些交易大部分是通过网络进行的。我们对美国四个主要的海洋水族馆鱼类电商销售平台进行了网络搜索, 确定了13个科的海洋辐鳍鱼(*Actinopterygii*)的零售价格和来源(野生捕捞、水产养殖或两者兼有)。我们还从FishBase数据库和世界自然保护联盟(IUCN)收集了这些鱼类的生态和经济特征数据作为补充。在所有平台和受欢迎的13个科的鱼类中, 我们发现有734个物种得到出售, 其中89.2%(655个物种)完全来自野生环境。45个物种受到保护关注(包括20个濒危物种和另外25个种群数量呈下降趋势的物种), 其中38个物种完全来自野生环境。零售价格与来源、体长、最小栖息深度和集群行为显著相关。此外, 有100个出售的物种在FishBase数据库或IUCN中没有被列为水族馆贸易物种, 表明这两个重要数据库中相关的渔业信息存在缺失。有58个物种(包括71个变种)同时有野生捕捞的个体和人工饲养的个体出售, 而水产养殖的个体比野生个体平均便宜28.1%(95%置信区间为15.3)。**【翻译:胡怡思;审校:聂永刚】**

## INTRODUCTION

The marine aquarium fish trade spans over 100 countries and involves more than 2000 species, the majority of which are wild caught to meet international demand (Miller‐Morgan, 2010; Rhyne, Tlusty, Szczebak, and Holmberg, 2017). In spite of its vast scale, the trade has consistently lagged behind other wildlife markets in research and policy attention (Scheffers et al., 2019; Smith et al., 2008). This gap was recently highlighted at a Convention on International Trade in Endangered Species (CITES) technical workshop, which emphasized the urgent need for better data on trade flows and the status of wild fish populations (CITES, 2024). Inadequate monitoring and limited regulatory oversight continue to obscure the trade's true extent, complicating efforts to assess its ecological impacts and develop effective management strategies (Biondo & Burki, 2020; Dee et al., 2014; Smith et al., 2008; Watson et al., 2023).

The United States has long played a pivotal role in the marine aquarium fish trade and currently absorbs nearly two‐thirds of global imports (Appendix A1) (Rhyne, Tlusty, Schofield et al., 2012; Rhyne, Tlusty, Szczebak, and Holmberg, 2017; Sinha et al., 2023). Much of this growth is associated with the rise of e‐commerce platforms, which began reshaping sales in the 2000s (Kay & Hoyle, 2001) but has not received commensurate attention from conservation researchers (Holmberg et al., 2015; Stringham et al., 2021).

The vast majority of species involved in the marine aquarium fish trade are sourced from the wild, which raises potential concerns for species overexploitation and ecosystem impacts (Palmtag, 2017). Some target species have elevated mortality rates throughout the supply chain (Miller‐Morgan, 2010). Unsustainable wild capture directly threatens species already facing elevated extinction risks and exacerbates other ecosystem‐ and species‐level stressors to wild populations (Tlusty, 2002). For instance, wild capture can affect nontarget species through incidental mortality and can cause ecosystem changes stemming from the removal of functionally important species (Rhyne et al., 2009).

The marine aquarium trade has also facilitated the accidental introduction of non‐native species across biogeographical boundaries (Padilla & Williams, 2004; Ruiz‐Allais et al., 2021). These introductions can result in species‐, population‐, and ecosystem‐level impacts and additional downstream consequences for the economies where invasive species are introduced (Holmberg et al., 2015; Lockwood et al., 2019; Padilla & Williams, 2004; Smith et al., 2008). Such consequences underscore the urgent need for better data on the nature of the trade, as well as on where regulatory measures and proactive conservation strategies are needed to mitigate the trade's deleterious effects on marine biodiversity and ecosystem integrity.

Against this backdrop, we assessed US online retail trade in popular marine aquarium ray‐finned fishes (*Actinopterygii*) to determine the extent to which the trade contributes to threats faced by marine finfishes. We also devised changes to the industry amidst growing conservation concerns.

## METHODS

To assess the state, scale, and scope of the online marine aquarium fish trade, we investigated 4 large saltwater fish dealers in the United States: Live Aquaria, Blue Zoo Aquatics, Saltwaterfish.com, and That Pet Place (see Appendices A1 & A6 for inclusion criteria and an interdealer species accumulation curve, respectively). From July 2021 to November 2024, we collected information on the taxonomic identity (variant, species, and family), dealer‐reported source (captive bred, wild caught, or both), geographic region of origin (if wild caught), and retail price for each fish variant for sale on each online e‐commerce platform. Search results were filtered to include only marine ray‐finned fish (*Actinopterygii*) and taxonomic families that had at least 25 unique species for sale or that contained captive‐bred species (see Appendices A2 & A3 for details on web scraping method and taxonomic inclusion criteria, respectively).

These restrictions allowed for the collection of representative data on pricing (derived from multiple e‐commerce platforms) and specimen source and provided a snapshot of demand‐and‐supply dynamics in the marine aquarium finfish market. Given these conservative inclusion criteria, our approach likely underestimated the absolute and relative abundance of wild‐caught fish for sale in the trade (because captive‐bred specimens were searched for specifically) and the total number of fish species, variants, and rare taxa available for sale across dealers.

For each recorded species, we extracted economic and ecological trait information from FishBase and the International Union for Conservation of Nature (IUCN), including maximum body length, occupied depth range, commercial importance and usage in the aquarium trade, diet, schooling behavior, extinction risk status, and life‐history traits (details in Appendix A4). For species‐level analyses, we used the median price of fish variants to control for phenotype‐based price variability and to minimize the influence of high‐priced outliers when testing associations with species‐level ecological and life‐history traits. Given the potential sensitivity of these data to COVID‐19 supply chain disruptions, we compared and validated our results with an external dataset of finfish retail prices collected from a mix of the same and different e‐commerce platforms in the United States from 2012 to 2013 (Holmberg et al., 2015) (details in Appendix A2).

To assess price correlates, we used a linear mixed‐effects model and Akaike information criterion corrected for small sample sizes (AICc) for model selection. Family and IUCN threat status were treated as random effects, and we evaluated the relative contributions (fixed effects) of each species’ maximum body length, diet, schooling behavior, geographic origin, minimum occupied ocean depth, occupied depth range, and source in an information‐theoretic framework (full model list in Appendix A9). For the species in our dataset that had both wild‐caught and captive‐bred specimens for sale, we used a paired *t* test to conduct price comparisons between the 2 sources. All analyses were conducted in R 4.3.2 with the *nlme* and MuMIn packages used to conduct the linear mixed‐effects modeling and AICc evaluations, respectively.

## RESULTS

**FIGURE 1:**
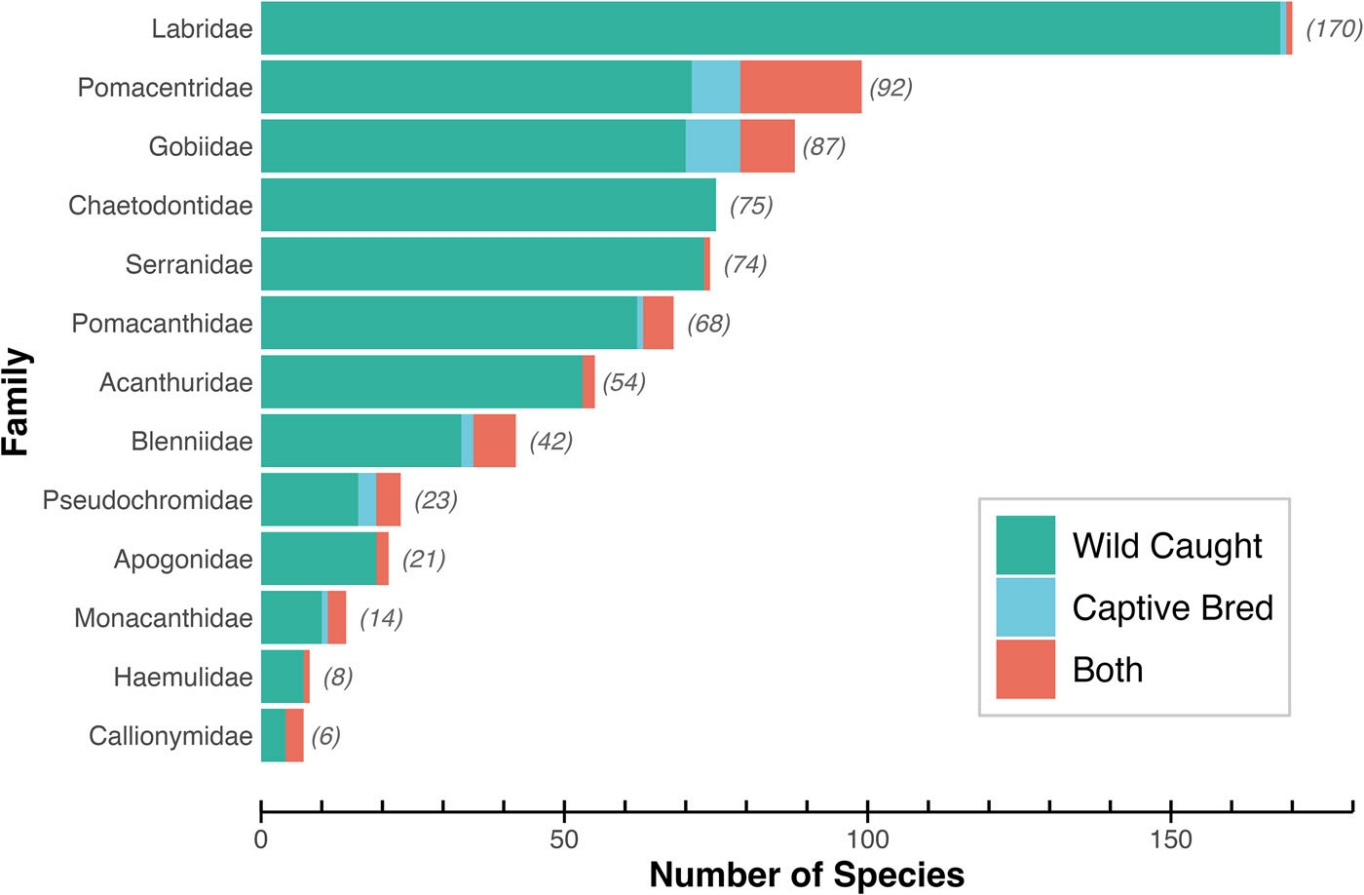
Thirteen ray‐finned fish (*Actinopterygii*) families and how they are sourced for online trade. Each family has ≥25 unique species or at least a single captive‐bred species for sale (parentheses indicate number of species for sale in each family). *Source*: Compare with Appendix S7.

We identified 734 fish species (encompassing 860 variants) that were traded across the 4 e‐commerce platforms among the 13 taxonomic families we assessed. Wrasses (*Labridae*) had the largest number of species for sale, followed by damselfishes (*Pomacentridae*) and gobies (*Gobiidae*) (Figure 1). Across all species, 89.2% (655 of 734) were sourced solely from the wild, 6.8% (50 of 734) were sourced from the wild and through aquaculture, 2.9% (21 of 734) were sourced exclusively through aquaculture, and 1.1% (8 of 734) had some variants that were wild caught and others that were obtained through aquaculture.

Of the 734 finfish species for sale, 45 were of conservation concern (i.e., classified as threatened or having decreasing population trends according to the IUCN). A total of 4 species were listed as endangered (*Pterapogon kauderni, Elacatinus prochilos, Gobiodon citrinus*, and *Amphiprion clarkii*), 16 were listed as vulnerable, and 33 had decreasing population trends (Appendix A8). Of these 45 species, 38 were sourced exclusively from the wild.

A total of 4 species were listed as near threatened (*Chaetodon rainfordi*, *Chaetodon trifascialis*, *Gobiodon ceramensis*, and *Pomacentrus lepidogenys*), 23 species had not yet been evaluated by the IUCN, 34 species were classified as data deficient (lacking sufficient information for an extinction risk assessment), and 463 species had unknown or unavailable population trend data. Notably, 100 species were not listed as in the aquarium trade on either the IUCN Red List or FishBase in spite of their presence on the e‐commerce platforms (Appendix A4).

**FIGURE 2:**
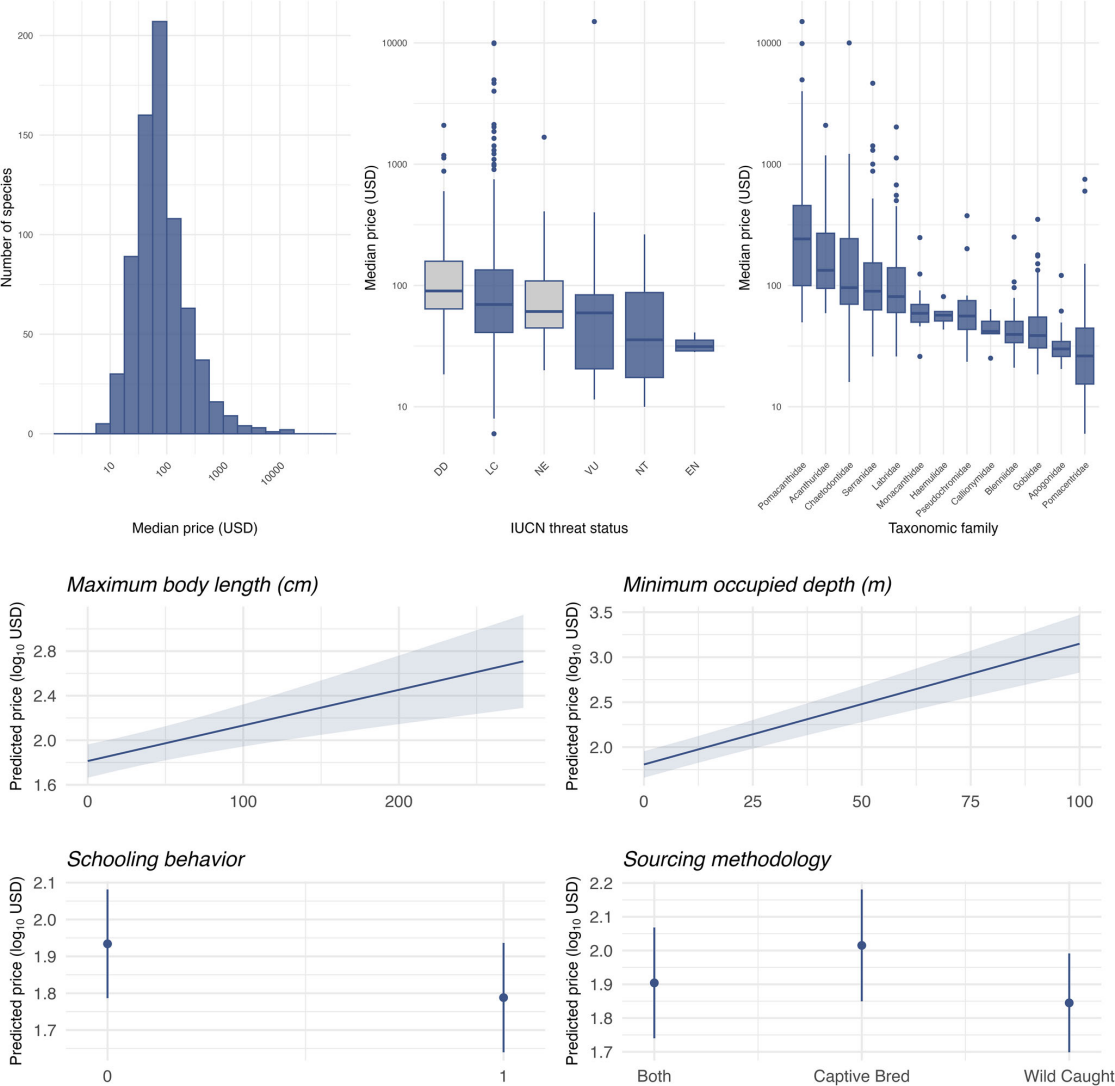
Median retail prices and price variability by International Union for Conservation of Nature (IUCN) threat status and taxonomic family (top) (random effects, taxonomic family and threat status) and marginal effects of fish maximum body length, minimum occupied depth, schooling behavior, and source on predicted prices (based on best mixed‐effects model) of ray‐finned fishes (*Actinopterygii*) traded online (bottom).

The mean price of a specimen across all 4 e‐commerce websites was $187.68 (95% confidence interval [CI] 51.66) (Figure 2). The median price was $66.30, and the 25% and 75% quantile prices were $39.99 and $129.99, respectively (Figure 2). Prices varied by threat status and taxonomic family (Figure 2 & Appendix A10).

Retail prices were significantly associated with fish source, maximum length, minimum occupied depth, and schooling behavior. This was indicated by the best performing model (ΔAICc = 0.00) from a 17‐candidate model list. Other well‐performing models included variables such as diet and geographic range (full list of candidate models, including a null model, is in Appendix A9). On average and across all species, traits such as being wild caught, having a short body length, occupying shallower depths, and exhibiting schooling behavior, were associated with lower retail prices (Figure 2).

**FIGURE 3:**
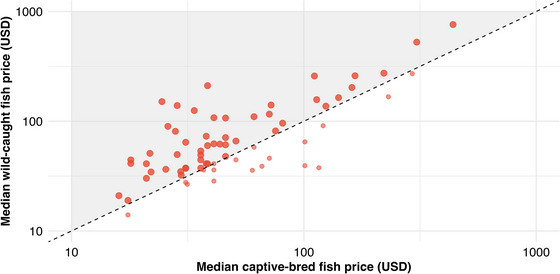
Prices for ray‐finned fishes (*Actinopterygii*) available online relative to their source as either captive bred or wild caught (shaded area, listings for which captive‐bred specimens were cheaper than wild‐caught specimens).

We also identified 71 variants (encompassing 58 species) in our dataset that had specimens sourced from both the wild and from aquaculture. When we compared the prices between these, we found that captive‐bred individuals were cheaper for 52 of the 71 variants analyzed. This difference was significant across all paired samples (*t* = 3.67, df = 70, *p* < 0.001) and led to a mean 28.12% (95% CI 15.26) reduction in price (Figure 3).

**FIGURE 4:**
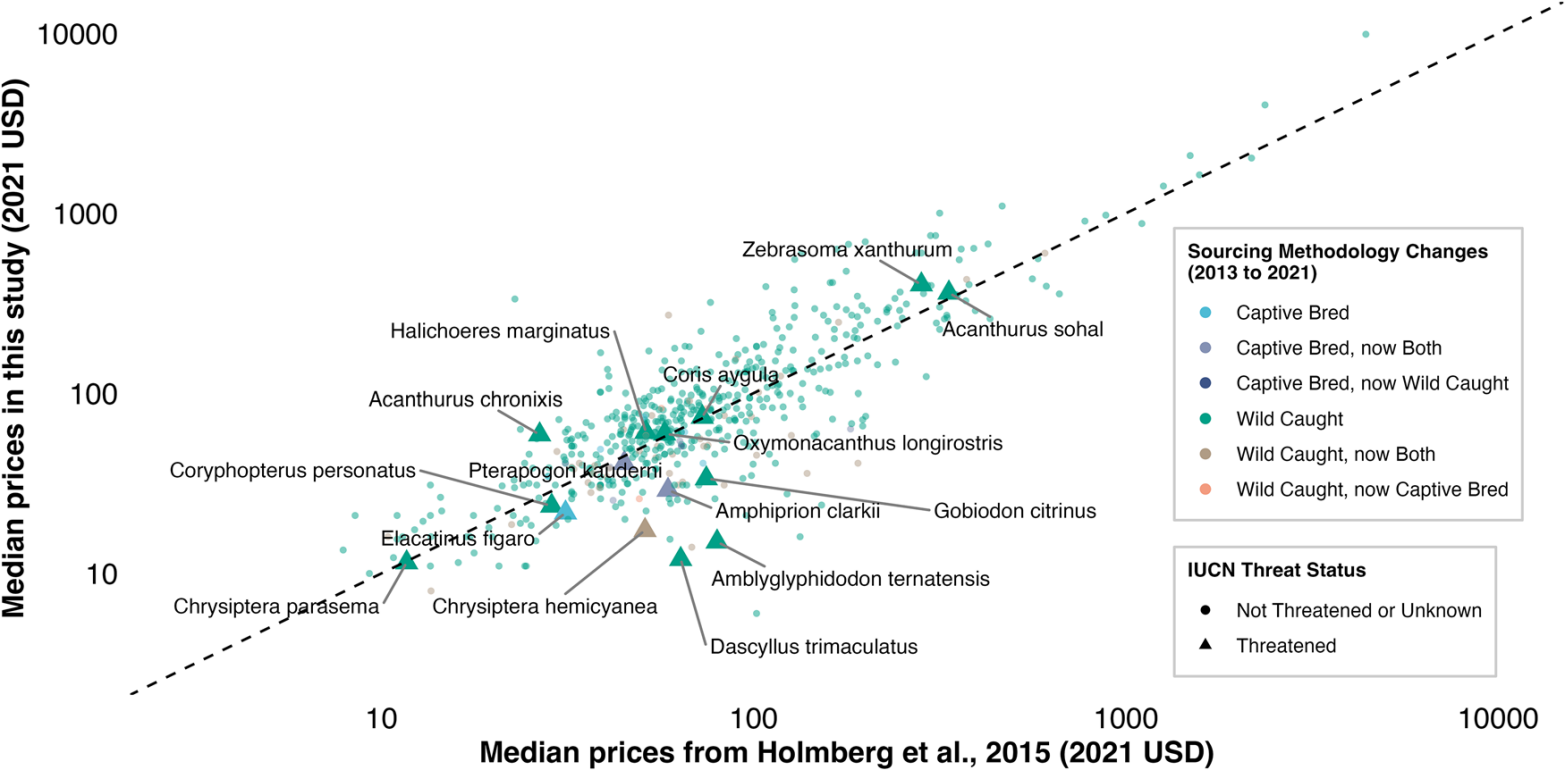
Changes in fish prices (adjusted to 2021 US dollars) and source from 2012–2013 to 2021–2024. Labeled species are those that are threatened according to the International Union for Conservation of Nature.

In the comparison of species available for sale in 2012–2013 and 2021–2024 (*n* = 556 species), inflation‐adjusted retail prices were highly correlated (*r* = 0.96, *p* < 0.001) (Figure 4) (differences between datasets in Appendix A11). Of these species, 88.7% (493 species) were reported as wild caught in both periods, and source remained largely consistent over time (Figure 4). Only *Elacatinus horsti* was primarily captive bred in 2013 and then primarily wild caught in 2021–2024. Three species, all clownfish (*Amphiprion barberi*, *Amphiprion ocellaris*, and *Amphiprion percula*), were wild caught in 2012–2013 and then captive bred in 2021–2024.

## DISCUSSION

Of the 734 species of marine fish we studied, 655 (89.2% of species) were sourced solely from the wild, and 100 were not recorded as being in the aquarium trade according to IUCN or FishBase, 2 authoritative databases of wild fish species. This heavy reliance on wild specimens, which is undoubtedly an underestimate given our methodology, could increase anthropogenic pressures in the habitats from which these hundreds of fish species are caught.

Typically, marine ornamental fish are captured in developing countries in the tropics, such as Indonesia and the Philippines (Palmtag, 2017), where their capture has historically (but not invariably) been linked to unsustainable or untargeted forms of fishing, including the use of cyanide (Dee et al., 2014; Rhyne, Tlusty, Schofield et al., 2012; Wood, 2001). Considering the documented high rates of direct and indirect mortality through the marine aquarium trade supply chain, sustained consumer demand will likely trigger additional and outsized levels of wild capture, especially in the absence of policy or community reforms around sustainable fisheries or viable alternative means of sourcing (Miller‐Morgan, 2010; Pountney, 2023; Watson et al., 2023). Evaluations, including productivity–susceptivity analyses, could help pinpoint specific species that are in need of greater oversight and management (Baillargeon et al., 2020).

Relying on sourcing information reported by online retailers requires caveats. Because these listings are based on self‐reported data and are not independently verified through certification or ecolabels, they are vulnerable to inadvertent errors and deliberate misrepresentation. Mislabeling of captive‐bred specimens—driven by taxonomic ambiguity and misidentification, consumer preferences, financial incentives, or efforts to launder wild‐caught individuals—has been well documented across various sectors of the wildlife trade and remains a pressing concern in the marine aquarium industry (Cohen et al., 2013; Esmail et al., 2020; Murray et al., 2012; Tlusty, 2002). However, in the absence of a centralized, publicly accessible system to track the provenance of marine aquarium fish, both in the United States and globally, online listings remain one of the few available ways to gain insight into this complex trade. We therefore interpreted reported sourcing designations with caution and underscore the urgent need for greater transparency and traceability throughout the global aquarium supply chain.

In lieu of sourcing from unsustainable wild fisheries, aquaculture may be a commercially viable path forward for conservation of traded fishes, especially for species already experiencing elevated levels of extinction risk (Rhyne, Tlusty, Schofield et al., 2012; Tlusty, 2002). Shifting to captive propagation could alleviate exploitation pressures placed on wild populations of target species, reduce bycatch of nontarget species, and mitigate ecological damages associated with destructive fishing methods. Due to the environmental and reproductive barriers that must be overcome for successful aquaculture, however, not all species are likely to be suitable for such strategies (Domínguez & Botella, 2014; Miller‐Morgan, 2010). Priority should be placed on marine fish with both high consumer demand and high extinction risk, and aquaculture efforts should be accompanied by behavioral interventions aimed at shifting consumer demand to sustainably sourced species, a process in which public aquariums could play an important role (Tlusty et al., 2013).

Such efforts should also not overlook the consequential impacts on local community suppliers that a switch to aquaculture might entail. This is especially important when desirable species become increasingly difficult to source in the wild because of overexploitation and reef degradation (Watson et al., 2023). Therefore promoting a sustainable marine aquarium trade in wild fish, possibly through carefully implemented certification schemes (Militz et al. 2017), may also be a viable option that is complementary to aquaculture. Careful monitoring of trends in the market price and volume of each species sold could be used to identify overexploited species, as has been suggested for the trade in wild‐caught songbirds (Harris et al., 2017). With all approaches, adequate information, adaptability, and oversight are key to ensuring the long‐term viability of reef ecosystems and the many communities that depend on them (Rhyne et al., 2014).

Additionally, the wild capture of fish for the marine aquarium trade can also drive socioeconomic resilience. In many coastal communities in exporting countries, this practice is a major source of local income and employment (Dee et al., 2014; Padilla & Williams, 2004; Rhyne et al., 2014; Rhyne, Tlusty, Szczebak, and Holmberg, 2017; Swanson et al., 2024). A shift entirely to aquaculture, which tends to operate as large‐scale enterprises in wealthier, importing countries, could disrupt traditional livelihoods and concentrate production and economic returns outside the communities and countries most in need of support (Smith et al., 2008; Stevens et al., 2017; Tlusty, 2002). Such outcomes would run contrary to many biodiversity conventions, which emphasize equitable benefit sharing and sustainable biodiversity use (CBD, 2017). These dynamics underscore the importance of balanced approaches that both protect marine biodiversity and safeguard socioeconomic benefits to vulnerable communities reliant on sustainable small‐scale fisheries.

We found that retail prices were strongly associated with multiple factors related to accessibility and ease of capture. Specifically, traits such as small body size, schooling behavior, and shallow depth ranges, were correlated with lower prices. When lower prices trigger commensurate increases in demand, this can put many ecologically similar species at risk, especially given the expansive demand for marine aquarium fish across multiple taxonomic families (Figure 1 & Appendix A7) (Rhyne, Tlusty, Schofield et al., 2012). Inevitable innovations in harvesting techniques or technologies will likely expand the suite of fish species accessible for capture, simultaneously reducing prices and raising demand.

Across all assessed species, our mixed‐effects models showed that wild‐caught species tended to be cheaper than captive‐bred species. This result might be driven by group living, wild‐caught species being easier to catch. This price difference could be problematic for conservation if wild caught, at‐risk species are readily accessible in the market. This finding also suggests that species’ extinction risks might be a market externality that is not fully and presently captured in online retail prices.

Conversely, for the 58 species in our dataset that had both captive‐bred and wild‐caught specimens for sale, pairwise comparisons showed aquaculture specimens were generally cheaper than wild‐caught specimens. This suggests that for some species, transitioning to aquaculture could help species conservation without hindering market supply or price. Additional factors in consumer preference and market demand could also drive this price differential, including the availability of diverse wild‐caught species acting as market competition (to reduce prices), the up‐front and overhead costs of establishing and maintaining aquaculture facilities, and the required research and development to commercially breed new species, especially in higher wage, developed countries (Watson et al., 2023). It has been observed that species new to aquaculture often command higher initial prices, which tend to fall as the species become more common (Rhyne, Tlusty, Kaufman, 2012).

In spite of the availability of aquaculture alternatives, wild‐caught fish often persist in the trade—likely reflecting a premium placed by hobbyists on traits such as phenotypic diversity, perceived resilience, and aesthetic appeal. Similar preferences have been documented for wild‐caught songbirds in Indonesia (Burivalova et al., 2017). This may help explain why only 25 species in our dataset were exclusively captive bred. For instance, the widely popular percula clownfish (*A. percula*) was listed at $149.99 for wild‐caught individuals and just $27.55 for captive‐bred individuals. Although this species is not currently threatened by collection (Maison & Graham, 2016), our dataset reflects only availability, not quantities sold, for this and other species (Rhyne, Tlusty, and Szczebak, 2017). For species vulnerable to overexploitation, aquaculture, coupled with effective fisheries management and sustainable harvesting practices, is likely essential to prevent further depletion (Baillargeon et al., 2020).

It is unusual that we detected no positive trend between price and rarity (operationalized by IUCN threat category) in our data (Figures 2 & 4). According to basic economic theory, prices typically rise as supply diminishes (Festa‐Bianchet, 2012), a pattern observed in other luxury wildlife markets (e.g., caviar: Gault et al., 2008). A study from Brazil showed higher online prices for prohibited fish (Borges et al., 2021), although this may reflect local market scarcity rather than true rarity in the wild. Perhaps in our data, and more broadly across the aquarium trade, the ready availability of rare and threatened species may be obscuring an unsustainable relationship between market price and species imperilment in the wild.

Irrespective of price, sustainable fisheries have the potential to play a crucial role in the marine aquarium trade by providing a means of sourcing fish without posing undue pressure on wild populations. Responsible management practices can help ensure that commonly traded fish species are harvested in ways that reduce bycatch, minimize habitat destruction, and maintain ecosystem equilibrium. Furthermore, sustainable fisheries can promote economic stability for local fishing communities and, when managed well, contribute to the conservation of both threatened species and the habitats on which they depend (Rhyne, Tlusty, Schofield et al., 2012; Tlusty, 2002; Watson et al., 2023).

The aquarium fish industry remains a high‐value yet understudied segment of the global wildlife trade—one that urgently demands greater transparency and scrutiny. A better understanding of the industry and its socioecological effects in the United States and globally will require rigorous assessments of trade economics across the supply chain, sustained monitoring of wild populations (especially for threatened species), and a clearer picture of ecological impacts where these fish originate. With this knowledge, policymakers can design evidence‐based regulations that safeguard not only vulnerable fish species but also the economic and environmental well‐being of coral reefs and the millions of people who depend on them.

## AUTHOR CONTRIBUTIONS

David S. Wilcove conceived the study with input from Bryan To, Bing Lin, and Yiwen Zeng. Bryan To conducted data collection with input from David S. Wilcove, Bing Lin, and Yiwen Zeng. Robert J. Holmberg provided historical data for comparative analyses. Bing Lin and Yiwen Zeng performed data analysis and drafted the manuscript. All authors contributed to subsequent manuscript versions.

## CONFLICT OF INTEREST STATEMENT

The authors declare no conflicts of interest.

## Supporting information



Supplementary Information

## Data Availability

Web scraped data and data extracted from the publicly accessible FishBase and IUCN databases are available from https://doi.org/10.5281/zenodo.10688436.
