# Supplementary material for: Extent of threats to marine fish from the online aquarium trade in the United States
Source: Conserv Biol. 2025 Oct 8;40(1):e70155. doi: 10.1111/cobi.70155 (PMC12856808; doi:10.1111/cobi.70155)
Supplement: Supplementary file 1 — Supplementary Information [file COBI-40-e70155-s001.docx]

Supplementary Information

# Appendix S1: Online dealer inclusion criteria

The United States stands out as a major market in the global marine aquarium fish trade, absorbing nearly two thirds of global trade (Sinha et al. 2023). It consistently tops international importation rankings, and plays a pivotal role in shaping global market trends (Smith et al. 2008; Rhyne et al. 2012, 2017). The US has held this dominant position for over two decades, driven in part by steady growth in demand for ornamental marine species since the 1990s, as home aquaria have become increasingly popular and accessible (Wabnitz 2003; Tissot et al. 2010; Rhyne et al. 2012; Dee et al. 2019). Much of this growth likely comes from the recent convenience and accessibility of e-commerce platforms, which began dominating sales as early as the 2000s (Kay & Hoyle 2001). However, these e-commerce platforms have attracted scant attention from conservation scientists to date (Holmberg et al. 2015). These factors, coupled with the often-opaque nature and structure of online markets, point to the outsized yet underexamined role of e-commerce platforms in the trade of marine fishes, in the US and everywhere (Stringham et al. 2021).

To evaluate aquarium dealers for study inclusion, we used a variety of Google search terms to find e-commerce platforms that were the most comprehensive and accessible to consumers in the US. Candidate search phrases included keywords such as “Aquarium”, “Saltwater”, and “Coral Reef”, which were paired with sale-related keywords such as “Trade”, “Dealer”, and “For Sale”. Our final search parameters were "Saltwater Coral Reef Fish For Sale", which produced a list of candidate dealers that were further narrowed to include e-commerce platforms that had saltwater fish for sale with accessible pricing information and navigable user interfaces. Private browsing modes were used to minimize possible location-dependent biases in search results.

These search parameters produced a list of ten prospective dealers in July 2021, whose total marine finfish inventories were manually tallied. At the time of counting (July 2021), Live Aquaria (<https://www.liveaquaria.com>) had ~950 marine finfish variants (phenotypes) for sale, That Pet Place (<https://www.thatpetplace.com>) also had ~950, Blue Zoo Aquatics (<https://www.bluezooaquatics.com>) had ~850, Salwaterfish.com (<https://www.saltwaterfish.com>) had ~800, Foxy Saltwater Tropicals (<https://mysaltwaterfishstore.com>) had ~350, Aquarium Creations Online (no longer active) had ~300, Manhattan Aquariums (<https://manhattanaquariums.com>) had ~250, KP Aquatics (<https://www.kpaquatics.com>) had ~150, Salty Underground (<https://www.saltyunderground.com>) had ~100, and Petco (<https://www.petco.com/shop/en/petcostore>) had ~50. Given the variation between the 10 candidate dealers, we proceeded with the top four dealers that were comparable in their breadth and volume of marine fish diversity sold: Live Aquaria, Blue Zoo Aquatics, Saltwaterfish.com, and That Pet Place.

As an additional benchmark to ensure that our chosen dealers were a fair representation of the scope of the US online ornamental marine fish trade, we created a rarefaction curve of unique species accrued as a function of each additional dealer we sampled. Wild-caught and captive-bred conspecifics, as well as intra-species phenotypic diversity, were pooled and treated as a single species (Appendix S6).

Of the 13 *Actinopterygii* families chosen for study inclusion, Blue Zoo Aquatics had 562 unique species for sale, Saltwaterfish.com had 517 unique species, Live Aquaria had 387 unique species, and That Pet Place had 369 unique species. Between dealers, this represents a tapering increase in unique species toa total of 734 unique species overall (Appendix S6). Importantly, these numbers neither reflect the total species or phenotypic inventory for each dealer, nor do they indicate the volume of specimens sold or available for each species.

# Appendix S2: Webscraping methodology

After selecting four online dealers, we manually visited each dealer's website to collect information on all available marine finfish phenotypes available for sale. For each variant listed, we recorded the following details: common name, Latin name, taxonomic family, sourcing method, aquaculture and/or wild-caught price (noting sale prices if applicable), origin (geographic region if wild-caught), length, and minimum and maximum occupied depth. The retail price for each fish from each dealer was calculated as the mean of all listed prices across different size categories and locations. For example, the price for *Chaetodon auriga* was averaged across small and three medium-sized listings, which represented slightly different geographic sources but not distinct phenotypic variations. In cases where distinct sizes (e.g., XS, S, M, L, XL) or multiple location-based prices (e.g., Philippines, Indonesia) were listed, the final price represents a mean across these categories.

Extinction risk, population trend, and use/trade data were obtained from IUCN in November 2023 and updated in August 2025. Species trait data were web scraped from FishBase in November–December 2023, and updated in August 2025, including: body shape, reef association, commercial importance, diet, habitat type, reported aquarium trade status, water column association, breeding structure, egg and larval distribution, sexual dimorphism, hermaphroditism, grouping tendencies, longevity, aggression level, diurnal or nocturnal behavior, and anemone association. Live Aquaria data were manually collected from August–September 2021. Blue Zoo Aquatics and Saltwaterifsh.com data were collected March–April 2022, and That Pet Place data were collected in July 2022. These data were supplemented by the inclusion of *Chaetodontidae* from all e-commerce platforms in November 2024 (paired *t* tests using the Wayback Machine were used to ensure that there were no significant price differences between these time frames.)

Additionally, our study was conducted during the COVID-19 pandemic, a period marked by global supply chain disruptions. These disruptions affected various aspects of species trade and pricing due to restricted availability, limited shipping capacities, and fluctuating demand patterns. This context likely influenced the observed price distributions in ways that may not reflect typical market conditions.

To account for COVID-19 supply chain disruptions that might cause our results to reflect anomalies related to the pandemic rather than consistent economic trends, we performed a sensitivity analysis on our data using a dataset collected from 2012–2013 (Holmberg et al. 2015). These data serve as a pre-COVID baseline for understanding trends within the marine aquarium trade and provide a point of comparison that allows us to validate our findings against pre-pandemic patterns.

Using Holmberg et al.’s data, we conducted pairwise comparisons between pre- and post-COVID 19 retail prices to validate that our findings reflect stable supply chain dynamics independent of the pandemic. This increased our confidence that observed trends in species pricing and sourcing were not dominated by COVID-related disruptions but rather reflect broader, more enduring economic patterns. We also analyzed shifts in the proportion of cultured versus wild-caught species between the two datasets. Holmberg et al.’s data provide a critical reference point for understanding sourcing practices within the trade, allowing us to track changes in the prevalence of cultured versus wild-caught species for sale over the past decade. This comparison highlights potential shifts in conservation practices, such as a move towards aquaculture, especially for species that are already of conservation concern (Figure 4)

# Appendix S3: Taxonomic family inclusion criteria

In this study, we limited our data collection to thirteen *Actinopterygii* families to ensure robust, representative pricing data that could be validated across multiple sellers, accurately reflecting supply-demand dynamics in the marine finfish trade. Our taxonomic family inclusion criteria focused on two main groups: finfish families with at least 25 unique species available for sale—Labridae (Wrasses), Pomacentridae (Clownfishes, Damselfishes, Chromis), Gobiidae (Gobies, Dartfishes, Firefishes), Chaetodontidae (Butterflyfishes), Serranidae (Anthias, Basslets, Basses, Groupers), Pomacanthidae (Angelfishes), Acanthuridae (Tangs), and Blenniidae (Blennies)—and families containing any number of cultured specimens—Apogonidae (Cardinalfishes), Callionymidae (Dragonets), Monacanthidae (Filefish), Haemulidae (Grunts, Sweetlips, Porkfish), and Pseudochromidae (Dottybacks). We observed a sharp drop in phenotypic diversity beyond these families, establishing a natural threshold for data inclusion. Other fish taxa (e.g., sharks and rays, *Chondrichthyes***)** and non-fish reef species, including invertebrates (e.g., stony corals, *Scleractinia*), were excluded from consideration.

We focused on families with aquacultured specimens to enable price comparisons between wild-caught and cultured conspecifics. Given that online dealers often categorize fish by common rather than scientific names (e.g., listing Clownfish, Damselfish, Angelfish, and Blennies separately), we manually sorted specimens by taxonomic family post-collection to ensure consistency. This selection likely also provides a conservative estimate of the conservation implications of the marine aquarium finfish trade given the underrepresentation of rare taxa under these data collection protocols.

# Appendix S4: FishBase and IUCN trait data

We extracted a host of ecological and economic fish trait data from both FishBase and IUCN. FishBase is an extensive database that provides extensive biological and ecological data on a wide array of fish species, including information on distribution, habitat preferences, population dynamics, and commercial uses. The IUCN maintains the Red List of Threatened Species, a global resource offering conservation status assessments for species based on consistently applied criteria such as population trends, habitat range, and extinction risk. From this list, we obtained each species’ extinction risk status (“Critically Endangered”, “Endangered”, “Vulnerable”, “Near Threatened”, and “Least Concern”) and population trend data (e.g., “Stable” or “Decreasing”). While extinction risk exists on a spectrum, we consider species classified by IUCN as Vulnerable, Endangered, and Critically Endangered by the IUCN as collectively “threatened” in this study.

For IUCN, we downloaded the "Range data - Points (CSV)" under the *Actinopterygii* criterion. We manually coded the “Habitat and Ecology” text into discrete fish life history traits. For FishBase, we downloaded the “Species” data from the “rfishbase” library in R and manually coded the text from the “Biology”, “Life Cycle and Mating Behavior”, and “Comments” sections into separate trait columns. We used the “Aquarium” column from FishBase and the “usetrade” dataset from the IUCN (subsetted for marine bony fishes) to determine whether species were designated as part of the aquarium trade in either source.

Across both FishBase and IUCN, traits included each species’ IUCN threat status, maximum length (cm), geographic origin (Pacific Ocean, Atlantic Ocean, Indian Ocean), minimum occupied depth (m), maximum occupied depth (m), vertical column location (pelagic, demersal benthopelagic, demersal benthic), eggs (pelagic, demersal), larvae (pelagic, not pelagic), diet (herbivore, carnivore, omnivore, detritivore), sexual dimorphism (binary), hermaphroditism (gonochoristic, simultaneous, protogynous, protandrous), grouping (singly, pairing, schooling), breeding (pair breeding, spawning aggregation, mouthbrooding), mating (monogamy, haremic, polygamy), territorial or aggression, longevity (years), diurnal or nocturnal, and anemone association (binary).

# Appendix S5: Correlates with retail prices

Linear mixed effects models were used to evaluate a total of 17 competing hypothetical models within an information theoretic framework (Appendix S9). These 17 models were selected based on a combination of availability of FishBase and IUCN trait data, and potential contributions of various species traits to retail price. Both fish family and IUCN threat status were treated as random effects in our study (see Appendix S10 for summary statistics). We used the following species traits as fixed effects based on their potential links to aquarium trade desirability, importation volume or price—maximum length, dietary preferences, schooling behavior, geography, minimum occupied depth, occupied depth range, and fish source (Winemiller, 2005; Smith et al. 2008; Rhyne et al. 2012). Model performance was evaluated with Akaike information criteria corrected for small sample sizes (Appendix S9).

# Appendix S6: Interdealer species accumulation curve


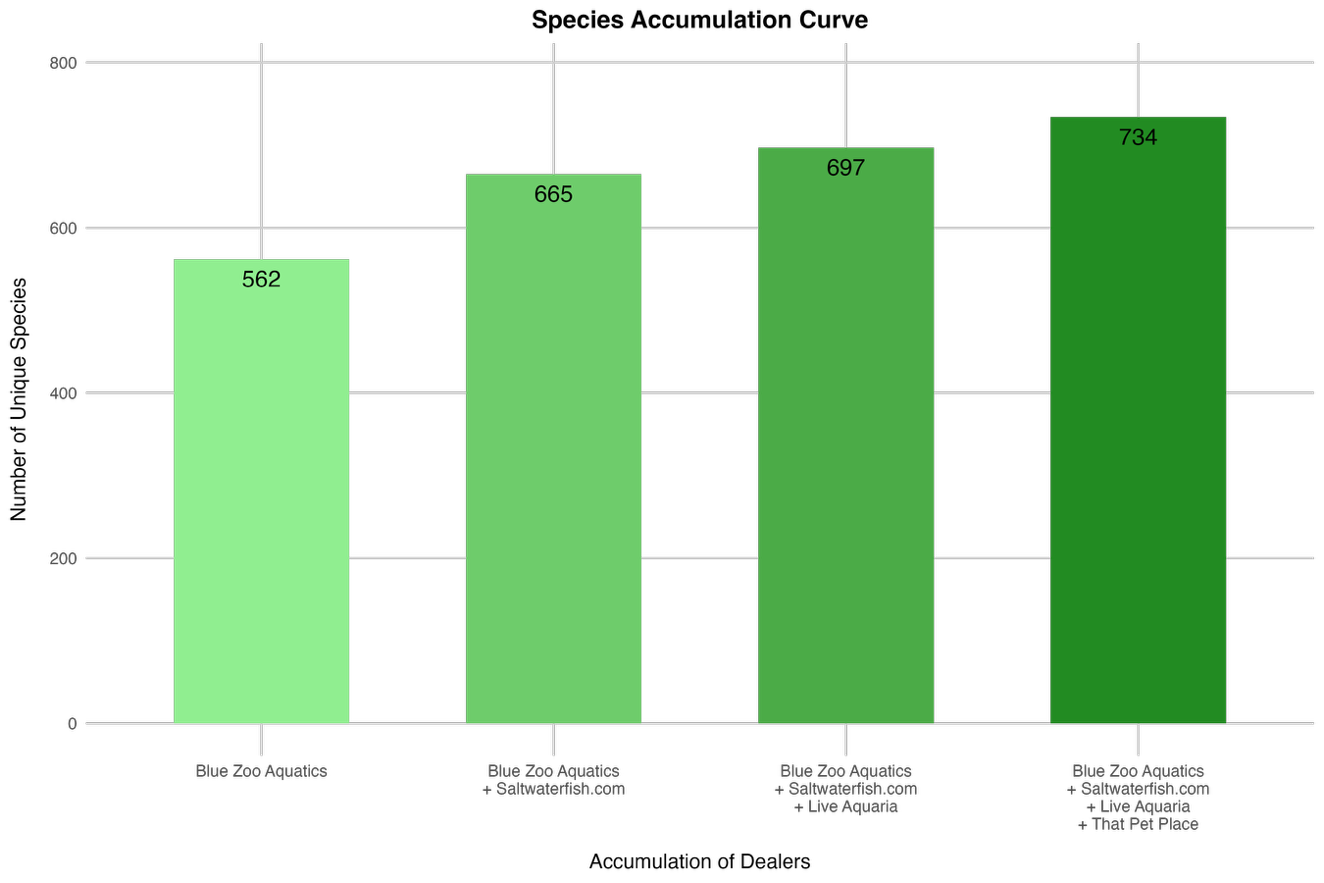


**Figure S6**: Species accumulation curve for the four marine aquarium trade dealers in this study

# Appendix S7: Finfish families with variants available for sale

**Figure S7**: Finfish species available for sale across all four e-commerce platforms, by taxonomic families. Light- and dark-blue colored bars represent taxonomic families included in the study.


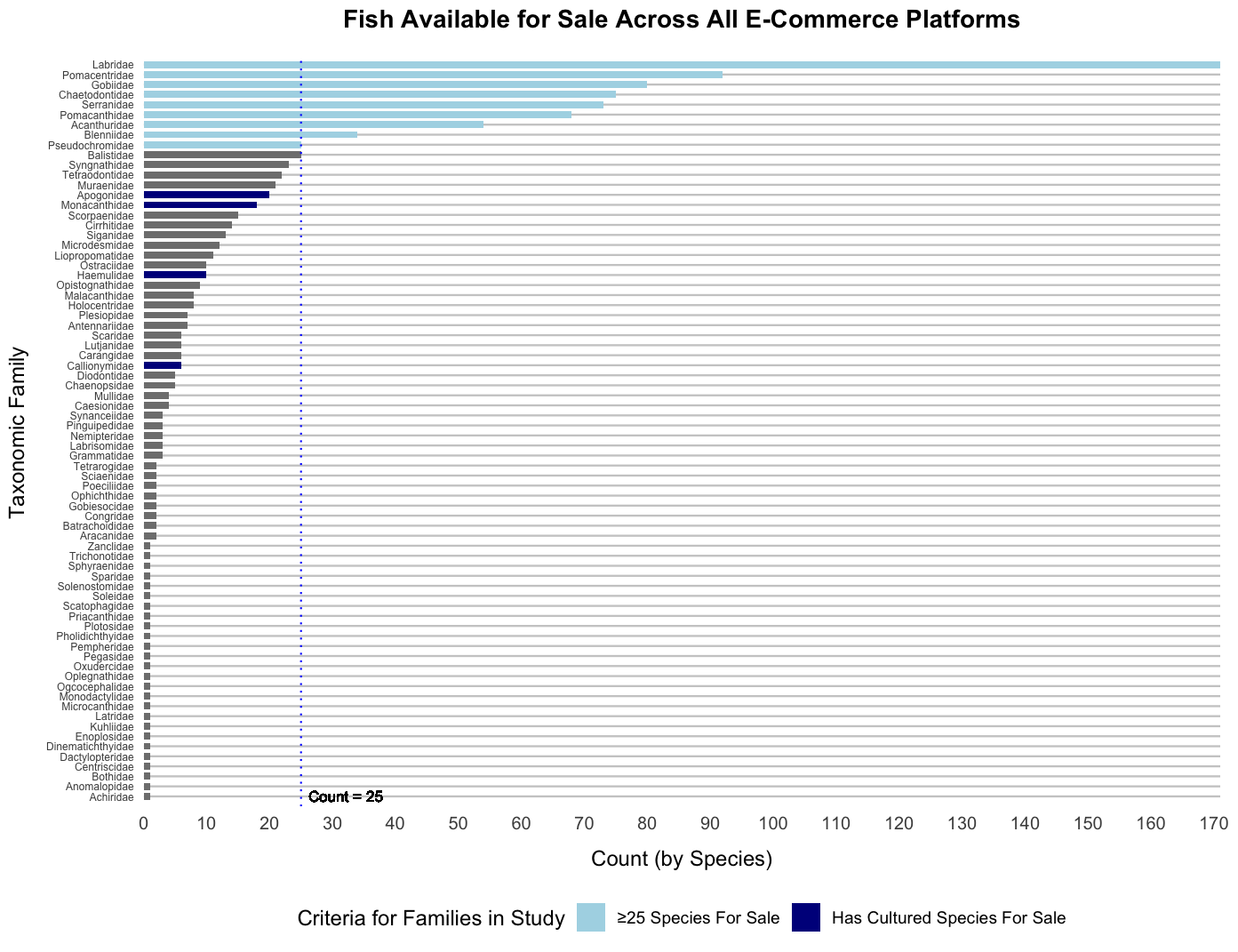


# Appendix S8: Imperiled species available for sale

| **Table S8:** Imperiled species available for sale | | | | |
| --- | --- | --- | --- | --- |
| Count | Species | Common Name | Source | IUCN Status |
| 1 | *Acanthurus chronixis* | Mimic Half Black Tang | Wild | VU |
| 2 | *Acanthurus sohal* | Sohal Tang | Wild | VU |
| 3 | *Zebrasoma xanthurum* | Purple Tang | Wild | VU |
| 4 | *Pterapogon kauderni* | Kaudern's Cardinalfish | Both | EN |
| 5 | *Ophioblennius steindachneri* | Panamic Fanged Blenny | Wild | VU |
| 6 | *Coryphopterus personatus* | Masked Goby | Wild | VU |
| 7 | *Elacatinus figaro* | Yellowline Goby | Cultured | VU |
| 8 | *Elacatinus prochilos* | Broadstripe Goby | Wild | EN |
| 9 | *Gobiodon citrinus* | Clown Goby, Citrinis | Wild | EN |
| 10 | *Oxymonacanthus longirostris* | Orangespotted Filefish | Wild | VU |
| 11 | *Lachnolaimus maximus* | Rooster Hogfish | Cultured | VU |
| 12 | *Coris aygula* | Twin Spot Wrasse | Wild | VU |
| 13 | *Halichoeres marginatus* | Dusky Wrasse | Wild | VU |
| 14 | *Holacanthus clarionensis* | Clarion Angelfish | Cultured | VU |
| 15 | *Amblyglyphidodon ternatensis* | Ternate Damselfish | Wild | VU |
| 16 | *Amphiprion clarkii* | Clarkii Clownfish | Both | EN |
| 17 | *Amphiprion mccullochi* | Tomato Clownfish | Cultured | VU |
| 18 | *Chrysiptera hemicyanea* | Azure Damselfish | Both | VU |
| 19 | *Chrysiptera parasema* | Yellowtail Damselfish | Wild | VU |
| 20 | *Dascyllus trimaculatus* | Three Spot Domino Damselfish | Wild | VU |

# Appendix S9: Full model list and performance evaluation

| **Table S9a:** Full model list for predictors of median price | | | | | |
| --- | --- | --- | --- | --- | --- |
| Model | Hypothesis | Fixed effect variables | df | AICc | ΔAICc |
| 15 | Price is affected by fish size, harvesting depth, schooling ability | maximum length, minimum occupied ocean depth, fish source, schooling behavior | 9 | 596.98 | 0.00 |
| 7 | Price is affected by fish size, harvesting depth, location, sourcing, schooling ability | maximum length, minimum occupied ocean depth, geography, fish source, schooling behavior | 11 | 598.07 | 1.10 |
| 9 | Price is affected by fish size, harvesting depth, sourcing, diet, schooling ability | maximum length, minimum occupied ocean depth, fish source, dietary preferences, schooling behavior | 11 | 600.55 | 3.57 |
| 1 | Price is affected by fish size, harvesting depth, location, sourcing, diet, schooling ability | maximum length, minimum occupied ocean depth, geography, fish source, dietary preferences, schooling behavior | 13 | 601.43 | 4.45 |
| 13 | Price is affected by fish size, harvesting depth sourcing | maximum length, minimum occupied ocean depth, fish source | 8 | 625.32 | 28.34 |
| 5 | Price is affected by fish size, harvesting depth, location, sourcing | maximum length, minimum occupied ocean depth, geography, fish source | 10 | 625.96 | 28.98 |
| 11 | Price is affected by fish size, harvesting depth, sourcing, diet | maximum length, minimum occupied ocean depth, fish source, dietary preferences | 10 | 626.41 | 29.43 |
| 3 | Price is affected by fish size, harvesting depth, location, sourcing, diet | maximum length, minimum occupied ocean depth, geography, fish source, dietary preferences | 12 | 626.86 | 29.88 |
| 8 | Price is affected by fish size, depth range, location, sourcing, schooling ability | maximum length, occupied depth range, geography, fish source, schooling behavior | 11 | 670.33 | 73.35 |
| 2 | Price is affected by fish size, depth range, sourcing, diet, schooling ability | maximum length, occupied depth range, geography, fish source, dietary preferences, schooling behavior | 13 | 671.86 | 74.88 |
| 16 | Price is affected by fish size, depth range, sourcing, schooling ability | maximum length, occupied depth range, fish source, schooling behavior | 9 | 674.09 | 77.11 |
| 10 | Price is affected by fish size, depth range, sourcing, diet, schooling ability | maximum length, occupied depth range, fish source, dietary preferences, schooling behavior | 11 | 675.82 | 78.84 |
| 17 | Null model | N/A | 4 | 686.07 | 89.09 |
| 4 | Price is affected by fish size, depth range, location, sourcing, diet | maximum length, occupied depth range, geography, fish source, dietary preferences | 12 | 703.03 | 106.05 |
| 6 | Price is affected by fish size, depth range, location, sourcing | maximum length, occupied depth range, geography, fish source | 10 | 704.49 | 107.51 |
| 12 | Price is affected by fish size, depth range, sourcing, diet | maximum length, occupied depth range, fish source, dietary preferences | 10 | 707.78 | 110.80 |
| 14 | Price is affected by fish size, depth range, sourcing | maximum length, occupied depth range, fish source | 8 | 709.06 | 112.08 |
| **Table S9b:** Full model list for predictors of mean price | | | | | |
| Model | Hypothesis | Fixed effect variables | df | AICc | ΔAICc |
| 15 | Price is affected by fish size, harvesting depth, schooling ability | maximum length, minimum occupied ocean depth, fish source, schooling behavior | 9 | 591.72 | 0.00 |
| 7 | Price is affected by fish size, harvesting depth, location, sourcing, schooling ability | maximum length, minimum occupied ocean depth, geography, fish source, schooling behavior | 11 | 593.69 | 1.97 |
| 9 | Price is affected by fish size, harvesting depth, sourcing, diet, schooling ability | maximum length, minimum occupied ocean depth, fish source, dietary preferences, schooling behavior | 11 | 594.99 | 3.27 |
| 1 | Price is affected by fish size, harvesting depth, location, sourcing, diet, schooling ability | maximum length, minimum occupied ocean depth, geography, fish source, dietary preferences, schooling behavior | 13 | 596.77 | 5.05 |
| 13 | Price is affected by fish size, harvesting depth sourcing | maximum length, minimum occupied ocean depth, fish source | 8 | 622.11 | 30.39 |
| 11 | Price is affected by fish size, harvesting depth, sourcing, diet | maximum length, minimum occupied ocean depth, fish source, dietary preferences | 10 | 622.78 | 31.06 |
| 5 | Price is affected by fish size, harvesting depth, location, sourcing | maximum length, minimum occupied ocean depth, geography, fish source | 10 | 623.44 | 31.72 |
| 3 | Price is affected by fish size, harvesting depth, location, sourcing, diet | maximum length, minimum occupied ocean depth, geography, fish source, dietary preferences | 12 | 623.96 | 32.24 |
| 8 | Price is affected by fish size, depth range, location, sourcing, schooling ability | maximum length, occupied depth range, geography, fish source, schooling behavior | 11 | 663.54 | 71.82 |
| 2 | Price is affected by fish size, depth range, sourcing, diet, schooling ability | maximum length, occupied depth range, geography, fish source, dietary preferences, schooling behavior | 13 | 664.84 | 73.12 |
| 16 | Price is affected by fish size, depth range, sourcing, schooling ability | maximum length, occupied depth range, fish source, schooling behavior | 9 | 666.37 | 74.65 |
| 10 | Price is affected by fish size, depth range, sourcing, diet, schooling ability | maximum length, occupied depth range, fish source, dietary preferences, schooling behavior | 11 | 667.84 | 76.12 |
| 17 | Null model | N/A | 4 | 682.08 | 90.36 |
| 4 | Price is affected by fish size, depth range, location, sourcing, diet | maximum length, occupied depth range, geography, fish source, dietary preferences | 12 | 697.88 | 106.16 |
| 6 | Price is affected by fish size, depth range, location, sourcing | maximum length, occupied depth range, geography, fish source | 10 | 699.68 | 107.96 |
| 12 | Price is affected by fish size, depth range, sourcing, diet | maximum length, occupied depth range, fish source, dietary preferences | 10 | 701.69 | 109.97 |
| 14 | Price is affected by fish size, depth range, sourcing | maximum length, occupied depth range, fish source | 8 | 703.36 | 111.64 |

# Appendix S10: Marine aquarium fish price summary statistics

| **Table S10a:** Marine aquarium fish price summary statistics by IUCN threat status | | | | | | | | |
| --- | --- | --- | --- | --- | --- | --- | --- | --- |
| IUCN status | Number of species | Mean | 95% CI | Min | Max | 25% quantile | Median | 75% quantile |
| Not Evaluated | 24 | 149.91 | 133.08 | 19.35 | 1668.79 | 44.32 | 67.13 | 103.99 |
| Data Deficient | 35 | 255.79 | 143.42 | 13.66 | 2090.99 | 62.99 | 92.99 | 154.99 |
| Least Concerned | 702 | 153.47 | 37.68 | 4.99 | 9882.49 | 37.70 | 63.64 | 120.37 |
| Near Threatened | 2 | 14.49 | 10.78 | 8.99 | 19.99 | 11.74 | 14.49 | 17.24 |
| Vulnerable | 17 | 962.27 | 1720.50 | 10.74 | 15000.00 | 16.87 | 57.57 | 73.66 |
| Endangered | 4 | 31.89 | 4.66 | 27.24 | 38.35 | 29.16 | 30.98 | 33.70 |

| **Table S10b:** Marine aquarium fish price summary statistics by taxonomic grouping | | | | | | | | |
| --- | --- | --- | --- | --- | --- | --- | --- | --- |
| Family | Number of species | Mean | 95% CI | Min | Max | 25% quantile | Median | 75% quantile |
| Acanthuridae | 58 | 233.23 | 79.53 | 58.12 | 2090.99 | 92.23 | 136.62 | 261.06 |
| Apogonidae | 21 | 35.23 | 9.26 | 19.49 | 119.99 | 24.99 | 29.44 | 33.49 |
| Blenniidae | 44 | 48.74 | 10.67 | 19.99 | 249.99 | 34.32 | 38.11 | 49.93 |
| Callionymidae | 8 | 49.31 | 13.14 | 24.64 | 88.88 | 40.67 | 46.08 | 52.47 |
| Gobiidae | 92 | 50.45 | 9.04 | 8.99 | 349.99 | 29.34 | 36.87 | 54.15 |
| Haemulidae | 8 | 59.18 | 8.12 | 42.32 | 79.99 | 51.74 | 59.52 | 62.43 |
| Labridae | 179 | 136.25 | 27.54 | 24.99 | 1939.55 | 59.91 | 81.56 | 145.71 |
| Monacanthidae | 15 | 75.28 | 28.88 | 24.99 | 247.16 | 47.26 | 57.49 | 67.49 |
| Pomacanthidae | 73 | 797.05 | 497.44 | 36.99 | 15000.00 | 96.52 | 234.32 | 412.72 |
| Pomacentridae | 189 | 63.74 | 14.17 | 4.99 | 898.99 | 20.49 | 39.99 | 74.32 |
| Pseudochromidae | 23 | 79.41 | 32.25 | 21.24 | 374.99 | 44.74 | 55.79 | 73.99 |
| Serranidae | 74 | 233.84 | 132.17 | 25.99 | 4649.99 | 62.53 | 90.43 | 158.13 |

# Appendix S11: Dataset comparison with Holmberg et al., 2015

| **Table S11a**: Species not in Holmberg et al., 2015 dataset (truncated to original 13 families) | | | |
| --- | --- | --- | --- |
| 1 | *Acanthurus bahianus* | 44 | *Heniochus singularius* |
| 2 | *Naso caeruleacauda* | 45 | *Prognathodes marcellae* |
| 3 | *Naso hexacanthus* | 46 | *Aioliops megastigma* |
| 4 | *Prionurus laticlavius* | 47 | *Amblyeleotris gymnocephala* |
| 5 | *Zebrasoma gemmatum* | 48 | *Amblyeleotris latifasciata* |
| 6 | *Apogon dovii* | 49 | *Amblyeleotris rubrimarginata* |
| 7 | *Apogon pseudomaculatus* | 50 | *Amblyeleotris yanoi* |
| 8 | *Apogon townsendi* | 51 | *Asterropteryx semipunctata* |
| 9 | *Apogonichthyoides melas* | 52 | *Cryptocentroides gobioides* |
| 10 | *Foa hyalina* | 53 | *Cryptocentrus cyanotaenia* |
| 11 | *Ostorhinchus aureus* | 54 | *Cryptocentrus fasciatus* |
| 12 | *Ostorhinchus hoevenii* | 55 | *Elacatinus illecebrosus* |
| 13 | *Ostorhinchus luteus* | 56 | *Elacatinus prochilos* |
| 14 | *Ostorhinchus margaritophorus* | 57 | *Eviota atriventris* |
| 15 | *Parapriacanthus ransonneti* | 58 | *Eviota nigriventris* |
| 16 | *Acanthemblemaria crockeri* | 59 | *Fusigobius neophytus* |
| 17 | *Acanthemblemaria hancocki* | 60 | *Fusigobius pallidus* |
| 18 | *Ecsenius nalolo* | 61 | *Gobiopsis quinquecincta* |
| 19 | *Ecsenius pictus* | 62 | *Istigobius decoratus* |
| 20 | *Ekemblemaria myersi* | 63 | *Lotilia graciliosa* |
| 21 | *Emblemaria pandionis* | 64 | *Pholidichthys leucotaenia* |
| 22 | *Enchelyurus flavipes* | 65 | *Priolepis aureoviridis* |
| 23 | *Gobioclinus kalisherae* | 66 | *Priolepis semidoliata* |
| 24 | *Istiblennius edentulus* | 67 | *Tigrigobius panamensis* |
| 25 | *Malacoctenus triangulatus* | 68 | *Trichonotus setiger* |
| 26 | *Meiacanthus kamoharai* | 69 | *Trimma benjamini* |
| 27 | *Ophioblennius steindachneri* | 70 | *Trimma caesiura* |
| 28 | *Paraclinus marmoratus* | 71 | *Trimma kitrinum* |
| 29 | *Plagiotremus rhinorhynchos* | 72 | *Trimma tevegae* |
| 30 | *Protemblemaria bicirrus* | 73 | *Valenciennea bella* |
| 31 | *Synchiropus sycorax* | 74 | *Valenciennea muralis* |
| 32 | *Chaetodon adiergastos* | 75 | *Gunnellichthys curiosus* |
| 33 | *Chaetodon blackburnii* | 76 | *Nemateleotris decora* |
| 34 | *Chaetodon madagaskariensis* | 77 | *Nemateleotris helfrichi* |
| 35 | *Chaetodon mesoleucos* | 78 | *Nemateleotris magnifica* |
| 36 | *Chaetodon nippon* | 79 | *Ptereleotris evides* |
| 37 | *Chaetodon robustus* | 80 | *Ptereleotris grammica* |
| 38 | *Chaetodon sedentarius* | 81 | *Ptereleotris hanae* |
| 39 | *Chaetodon trifascialis* | 82 | *Ptereleotris heteroptera* |
| 40 | *Coradion chrysozonus* | 83 | *Ptereleotris microlepis* |
| 41 | *Heniochus chrysostomus* | 84 | *Ptereleotris zebra* |
| 42 | *Heniochus monoceros* | 85 | *Diagramma pictum* |
| 43 | *Heniochus pleurotaenia* | 86 | *Haemulon flavolineatum* |
| 87 | *Acreichthys radiatus* | 133 | *Novaculops halsteadi* |
| 88 | *Cantherhines macrocerus* | 134 | *Belonoperca chabanaudi* |
| 89 | *Cantherhines pullus* | 135 | *Belonoperca pylei* |
| 90 | *Monacanthus chinensis* | 136 | *Cephalopholis cruentata* |
| 91 | *Monacanthus ciliatus* | 137 | *Dermatolepis inermis* |
| 92 | *Pseudalutarius nasicornis* | 138 | *Diplectrum formosum* |
| 93 | *Rudarius ercodes* | 139 | *Epinephelus fasciatus* |
| 94 | *Stephanolepis hispida* | 140 | *Epinephelus guttatus* |
| 95 | *Bodianus diplotaenia* | 141 | *Epinephelus ongus* |
| 96 | *Bodianus neopercularis* | 142 | *Epinephelus spilotoceps* |
| 97 | *Lachnolaimus maximus* | 143 | *Hypoplectrus nigricans* |
| 98 | *Anampses caeruleopunctatus* | 144 | *Hypoplectrus puella* |
| 99 | *Anampses lineatus* | 145 | *Hypoplectrus unicolor* |
| 100 | *Cetoscarus bicolor* | 146 | *Liopropoma fasciatum* |
| 101 | *Choerodon anchorago* | 147 | *Liopropoma mowbrayi* |
| 102 | *Clepticus parrae* | 148 | *Liopropoma multilineatum* |
| 103 | *Hologymnosus doliatus* | 149 | *Luzonichthys waitei* |
| 104 | *Hologymnosus rhodonotus* | 150 | *Luzonichthys whitleyi* |
| 105 | *Scarus taeniopterus* | 151 | *Plectropomus laevis* |
| 106 | *Thalassoma trilobatum* | 152 | *Pseudanthias cooperi* |
| 107 | *Cirrhilabrus beauperryi* | 153 | *Pseudanthias engelhardi* |
| 108 | *Cirrhilabrus brunneus* | 154 | *Pseudanthias hutomoi* |
| 109 | *Cirrhilabrus cyanogularis* | 155 | *Pseudogramma xantha* |
| 110 | *Cirrhilabrus isosceles* | 156 | *Sacura margaritacea* |
| 111 | *Cirrhilabrus johnsoni* | 157 | *Serranus pulcher* |
| 112 | *Cirrhilabrus katherinae* | 158 | *Centropyge deborae* |
| 113 | *Cirrhilabrus katoi* | 159 | *Centropyge woodheadi* |
| 114 | *Cirrhilabrus marjorie* | 160 | *Apolemichthys arcuatus* |
| 115 | *Cirrhilabrus melanomarginatus* | 161 | *Apolemichthys armitagei* |
| 116 | *Cirrhilabrus nahackyi* | 162 | *Apolemichthys kingi* |
| 117 | *Cirrhilabrus rubeus* | 163 | *Apolemichthys xanthotis* |
| 118 | *Coris pictoides* | 164 | *Chaetodontoplus personifer* |
| 119 | *Halichoeres bicolor* | 165 | *Holacanthus clarionensis* |
| 120 | *Halichoeres chierchiae* | 166 | *Amblypomacentrus breviceps* |
| 121 | *Halichoeres cyanocephalus* | 167 | *Amphiprion mccullochi* |
| 122 | *Halichoeres maculipinna* | 168 | *Azurina atrilobata* |
| 123 | *Halichoeres margaritaceus* | 169 | *Azurina lepidolepis* |
| 124 | *Halichoeres melasmapomus* | 170 | *Chromis limbata* |
| 125 | *Halichoeres nebulosus* | 171 | *Chromis nitida* |
| 126 | *Halichoeres timorensis* | 172 | *Chrysiptera notialis* |
| 127 | *Macropharyngodon moyeri* | 173 | *Chrysiptera rex* |
| 128 | *Paracheilinus attenuatus* | 174 | *Chrysiptera traceyi* |
| 129 | *Pseudocoris yamashiroi* | 175 | *Dischistodus perspicillatus* |
| 130 | *Pseudojuloides mesostigma* | 176 | *Microspathodon dorsalis* |
| 131 | *Pteragogus enneacanthus* | 11777 | *Plectroglyphidodon johnstonianus* |
| 132 | *Xenojulis margaritacea* | 178 | *Novaculops halsteadi* |
| 179 | *Pomacentrus amboinensis* |  | |
| 180 | *Pomacentrus caeruleus* |  |  |
| 181 | *Pomacentrus chrysurus* |  |  |
| 182 | *Pomacentrus lepidogenys* |  |  |
| 183 | *Pomacentrus pavo* |  |  |
| 184 | *Pomacentrus sulfureus* |  |  |
| 185 | *Stegastes leucostictus* |  |  |
| 186 | *Stegastes planifrons* |  |  |
| 187 | *Pseudochromis caudalis* |  |  |
| 188 | *Pseudoplesiops typus* |  |  |

| **Table S11b**: Species present in Holmberg et al., 2015 but not in this dataset | | | |
| --- | --- | --- | --- |
| 1 | *Pycnochromis acares* | 20 | *Pyronotanthias parvirostris* |
| 2 | *Pycnochromis agilis* | 21 | *Pseudochromis bitaeniatus* |
| 3 | *Pycnochromis margaritifer* | 22 | *Cephalopholis taeniops* |
| 4 | *Chromis opercularis* | 23 | *Pycnochromis amboinensis* |
| 5 | *Chromis xanthura* | 24 | *Amblypomacentrus tricinctus* |
| 6 | *Congrogadus subducens* | 25 | *Hypsypops rubicundus* |
| 7 | *Coryphopterus glaucofraenum* | 26 | *Mirolabrichthys tuka* |
| 8 | *Chromileptes altivelis* | 27 | *Pycnochromis dimidiatus* |
| 9 | *Dascyllus carneus* | 28 | *Apogon erythrinus* |
| 10 | *Dascyllus flavicaudus* | 29 | *Brachygobius doriae* |
| 11 | *Plectorhinchus centurio* | 30 | *Chaetodon auripes* |
| 12 | *Halichoeres claudia* | 31 | *Grammistes sexlineatus* |
| 13 | *Pictichromis dinar* | 32 | *Istiblennius zebra* |
| 14 | *Plectorhinchus lineatus* | 33 | *Pyronotanthias flavoguttatus* |
| 15 | *Plectropomus maculatus* | 34 | *Pyronotanthias lori* |
| 16 | *Nemanthias bartlettorum* | 35 | *Stegastes diencaeus* |
| 17 | *Nemanthias bicolor* | 36 | *Apogon binotatus* |
| 18 | *Nemanthias dispar* | 37 | *Apogon crassiceps* |
| 19 | *Mirolabrichthys evansi* | 38 | *Halichoeres pictus* |

# Appendix S12: Temporal source change comparison

| **Table S12**: Matched dataset from Holmberg et al., 2015 to this study | | | | | |
| --- | --- | --- | --- | --- | --- |
|  | Species | IUCN (2025) | Price (USD)  (Lin et al., 2025) | Price (USD)  (Holmberg et al., 2015) | Source (Change) |
| 1 | Abudefduf saxatilis | LC | 8.99 | 6.57 | Wild-Caught |
| 2 | Acanthochromis polyacanthus | LC | 9.99 | 18.47 | Wild-Caught |
| 3 | Acanthurus achilles | LC | 629.99 | 131.98 | Wild-Caught |
| 4 | Acanthurus bariene | LC | 363.63 | 195.51 | Wild-Caught |
| 5 | Acanthurus blochii | LC | 82.33 | 88.28 | Wild-Caught |
| 6 | Acanthurus chirurgus | LC | 82.49 | 49.96 | Wild-Caught |
| 7 | Acanthurus chronixis | VU | 58.12 | 20.32 | Wild-Caught |
| 8 | Acanthurus coeruleus | LC | 98.74 | 50.24 | Wild-Caught |
| 9 | Acanthurus dussumieri | LC | 391.88 | 484.24 | Wild-Caught |
| 10 | Acanthurus fowleri | LC | 430.66 | 444.08 | Wild-Caught |
| 11 | Acanthurus guttatus | LC | 410.53 | 217.03 | Wild-Caught |
| 12 | Acanthurus japonicus | LC | 104.61 | 44.11 | Wild-Caught |
| 13 | Acanthurus leucocheilus | LC | 286.33 | 311.84 | Wild-Caught |
| 14 | Acanthurus leucopareius | LC | 261.99 | 261.95 | Wild-Caught |
| 15 | Acanthurus leucosternon | LC | 128.82 | 71.29 | Wild-Caught |
| 16 | Acanthurus lineatus | LC | 95.51 | 62.70 | Wild-Caught |
| 17 | Acanthurus maculiceps | LC | 258.26 | 341.62 | Wild-Caught |
| 18 | Acanthurus mata | LC | 101.66 | 122.95 | Wild-Caught |
| 19 | Acanthurus monroviae | LC | 224.99 | 249.95 | Wild-Caught |
| 20 | Acanthurus nigricans | LC | 170.42 | 40.70 | Wild-Caught |
| 21 | Acanthurus nigricauda | LC | 134.99 | 119.09 | Wild-Caught |
| 22 | Acanthurus nigrofuscus | LC | 79.99 | 44.56 | Wild-Caught |
| 23 | Acanthurus nigroris | LC | 83.33 | 83.28 | Wild-Caught |
| 24 | Acanthurus nubilus | LC | 63.33 | 63.28 | Wild-Caught |
| 25 | Acanthurus olivaceus | LC | 111.91 | 69.98 | Wild-Caught |
| 26 | Acanthurus pyroferus | LC | 90.20 | 46.71 | Wild-Caught |
| 27 | Acanthurus sohal | VU | 359.83 | 264.68 | Wild-Caught |
| 28 | Acanthurus tennentii | LC | 126.66 | 55.48 | Wild-Caught |
| 29 | Acanthurus thompsoni | LC | 109.16 | 88.30 | Wild-Caught |
| 30 | Acanthurus triostegus | LC | 61.05 | 48.51 | Wild-Caught |
| 31 | Acanthurus tristis | LC | 106.79 | 54.95 | Wild-Caught |
| 32 | Acreichthys tomentosus | LC | 58.49 | 38.47 | Wild-Caught, now Both |
| 33 | Amblyeleotris aurora | LC | 59.99 | 42.21 | Wild-Caught |
| 34 | Amblyeleotris diagonalis | LC | 26.66 | 96.77 | Wild-Caught |
| 35 | Amblyeleotris guttata | LC | 29.99 | 31.68 | Wild-Caught |
| 36 | Amblyeleotris randalli | LC | 58.33 | 34.14 | Wild-Caught |
| 37 | Amblyeleotris steinitzi | LC | 21.99 | 15.47 | Wild-Caught |
| 38 | Amblyeleotris wheeleri | LC | 29.66 | 15.95 | Wild-Caught |
| 39 | Amblyglyphidodon aureus | LC | 14.16 | 19.84 | Wild-Caught |
| 40 | Amblyglyphidodon curacao | LC | 11.99 | 13.28 | Wild-Caught |
| 41 | Amblyglyphidodon ternatensis | VU | 13.99 | 62.49 | Wild-Caught |
| 42 | Amblygobius decussatus | LC | 18.99 | 18.63 | Wild-Caught |
| 43 | Amblygobius phalaena | LC | 46.62 | 22.71 | Wild-Caught, now Both |
| 44 | Amblygobius semicinctus | LC | 62.24 | 17.95 | Wild-Caught |
| 45 | Amblypomacentrus tricinctus | NE | 44.99 | 24.95 | Wild-Caught |
| 46 | Amphiprion akallopisos | LC | 54.16 | 24.20 | Wild-Caught |
| 47 | Amphiprion akindynos | LC | 73.32 | 77.47 | Wild-Caught, now Both |
| 48 | Amphiprion allardi | LC | 57.32 | 35.85 | Wild-Caught |
| 49 | Amphiprion barberi | NE | 24.99 | 38.32 | Wild-Caught, now Captive-Bred |
| 50 | Amphiprion biaculeatus | LC | 62.04 | 34.70 | Wild-Caught, now Both |
| 51 | Amphiprion bicinctus | LC | 62.49 | 143.71 | Captive-Bred, now Both |
| 52 | Amphiprion chrysopterus | LC | 50.62 | 59.04 | Wild-Caught, now Both |
| 53 | Amphiprion clarkii | EN | 28.09 | 45.93 | Captive-Bred, now Both |
| 54 | Amphiprion ephippium | LC | 37.99 | 31.83 | Captive-Bred, now Both |
| 55 | Amphiprion frenatus | LC | 27.16 | 28.20 | Wild-Caught, now Both |
| 56 | Amphiprion latezonatus | DD | 598.74 | 479.95 | Wild-Caught, now Both |
| 57 | Amphiprion leucokranos | LC | 749.99 | 243.70 | Wild-Caught |
| 58 | Amphiprion melanopus | LC | 31.24 | 50.33 | Wild-Caught, now Both |
| 59 | Amphiprion nigripes | LC | 42.99 | 42.53 | Wild-Caught, now Both |
| 60 | Amphiprion ocellaris | LC | 81.79 | 44.04 | Wild-Caught, now Captive-Bred |
| 61 | Amphiprion percula | LC | 81.32 | 58.47 | Wild-Caught, now Captive-Bred |
| 62 | Amphiprion perideraion | LC | 29.99 | 48.91 | Wild-Caught, now Both |
| 63 | Amphiprion polymnus | LC | 33.41 | 31.19 | Wild-Caught, now Both |
| 64 | Amphiprion rubrocinctus | LC | 31.66 | 31.62 | Captive-Bred |
| 65 | Amphiprion sandaracinos | LC | 30.99 | 27.70 | Wild-Caught, now Both |
| 66 | Amphiprion sebae | LC | 32.24 | 31.42 | Wild-Caught |
| 67 | Amphiprion tricinctus | LC | 108.87 | 91.20 | Wild-Caught |
| 68 | Anampses chrysocephalus | LC | 104.24 | 61.20 | Wild-Caught |
| 69 | Anampses femininus | LC | 2024.99 | 1724.95 | Wild-Caught |
| 70 | Anampses melanurus | LC | 29.99 | 29.95 | Wild-Caught |
| 71 | Anampses meleagrides | LC | 75.19 | 34.95 | Wild-Caught |
| 72 | Anampses neoguinaicus | LC | 98.99 | 35.15 | Wild-Caught |
| 73 | Anampses twistii | LC | 44.99 | 86.63 | Wild-Caught |
| 74 | Anisotremus virginicus | LC | 49.99 | 42.95 | Wild-Caught, now Both |
| 75 | Apogon maculatus | LC | 28.99 | 17.28 | Wild-Caught |
| 76 | Apolemichthys griffisi | LC | 902.99 | 614.95 | Wild-Caught |
| 77 | Apolemichthys trimaculatus | LC | 132.26 | 100.73 | Wild-Caught |
| 78 | Apolemichthys xanthopunctatus | LC | 549.99 | 282.24 | Wild-Caught |
| 79 | Apolemichthys xanthurus | LC | 74.49 | 41.47 | Wild-Caught, now Both |
| 80 | Atrosalarias fuscus | LC | 43.12 | 36.74 | Wild-Caught |
| 81 | Azurina cyanea | LC | 48.12 | 65.47 | Wild-Caught |
| 82 | Blenniella chrysospilos | LC | 49.99 | 43.98 | Wild-Caught |
| 83 | Bodianus anthioides | LC | 131.64 | 75.68 | Wild-Caught |
| 84 | Bodianus axillaris | LC | 52.49 | 46.90 | Wild-Caught |
| 85 | Bodianus bilunulatus | LC | 48.19 | 40.15 | Wild-Caught |
| 86 | Bodianus bimaculatus | LC | 71.66 | 69.62 | Wild-Caught |
| 87 | Bodianus diana | LC | 68.66 | 43.03 | Wild-Caught |
| 88 | Bodianus loxozonus | LC | 29.99 | 29.95 | Wild-Caught |
| 89 | Bodianus masudai | LC | 224.99 | 129.91 | Wild-Caught |
| 90 | Bodianus mesothorax | LC | 72.49 | 95.07 | Wild-Caught |
| 91 | Bodianus pulchellus | LC | 147.74 | 82.56 | Wild-Caught |
| 92 | Bodianus rufus | LC | 91.24 | 60.47 | Wild-Caught |
| 93 | Bodianus sepiacaudus | DD | 169.49 | 119.95 | Wild-Caught |
| 94 | Bodianus speciosus | DD | 129.99 | 74.95 | Wild-Caught |
| 95 | Centropyge acanthops | LC | 99.99 | 80.98 | Wild-Caught, now Both |
| 96 | Centropyge argi | LC | 54.99 | 24.97 | Wild-Caught |
| 97 | Centropyge aurantia | LC | 289.99 | 179.95 | Wild-Caught |
| 98 | Centropyge aurantonotus | LC | 279.99 | 88.22 | Wild-Caught |
| 99 | Centropyge bicolor | LC | 68.99 | 42.51 | Wild-Caught |
| 100 | Centropyge bispinosa | LC | 74.37 | 65.42 | Wild-Caught, now Both |
| 101 | Centropyge colini | LC | 599.99 | 223.28 | Wild-Caught |
| 102 | Centropyge eibli | LC | 68.74 | 40.11 | Wild-Caught |
| 103 | Centropyge ferrugata | LC | 55.44 | 27.20 | Wild-Caught |
| 104 | Centropyge fisheri | LC | 56.88 | 66.83 | Wild-Caught |
| 105 | Centropyge flavipectoralis | LC | 48.74 | 43.68 | Wild-Caught |
| 106 | Centropyge flavissima | LC | 112.18 | 47.75 | Wild-Caught |
| 107 | Centropyge heraldi | LC | 56.87 | 41.67 | Wild-Caught |
| 108 | Centropyge interrupta | LC | 3999.99 | 1874.95 | Wild-Caught |
| 109 | Centropyge joculator | LC | 1633.33 | 1249.95 | Wild-Caught |
| 110 | Centropyge loriculus | LC | 116.12 | 67.27 | Wild-Caught |
| 111 | Centropyge multicolor | LC | 123.82 | 83.06 | Wild-Caught |
| 112 | Centropyge multispinis | LC | 79.99 | 60.06 | Wild-Caught |
| 113 | Centropyge nox | LC | 51.24 | 49.98 | Wild-Caught |
| 114 | Centropyge potteri | LC | 185.49 | 83.56 | Wild-Caught |
| 115 | Centropyge shepardi | LC | 399.99 | 299.95 | Wild-Caught |
| 116 | Centropyge tibicen | LC | 70.66 | 22.99 | Wild-Caught |
| 117 | Centropyge venusta | LC | 221.66 | 147.88 | Wild-Caught |
| 118 | Centropyge vrolikii | LC | 54.49 | 39.85 | Wild-Caught |
| 119 | Cephalopholis argus | LC | 139.03 | 98.93 | Wild-Caught |
| 120 | Cephalopholis boenak | LC | 162.78 | 71.20 | Wild-Caught |
| 121 | Cephalopholis formosa | LC | 99.99 | 126.66 | Wild-Caught |
| 122 | Cephalopholis fulva | LC | 76.66 | 76.62 | Wild-Caught |
| 123 | Cephalopholis miniata | LC | 137.99 | 75.38 | Wild-Caught |
| 124 | Cephalopholis polleni | LC | 259.99 | 198.21 | Wild-Caught |
| 125 | Cephalopholis urodeta | LC | 70.39 | 39.66 | Wild-Caught |
| 126 | Cetoscarus bicolor | LC | 31.66 | 32.37 | Wild-Caught |
| 127 | Chaetodermis penicilligerus | LC | 123.32 | 92.96 | Wild-Caught, now Both |
| 128 | Chaetodon argentatus | LC | 53.33 | 36.62 | Wild-Caught |
| 129 | Chaetodon auriga | LC | 84.74 | 55.82 | Wild-Caught |
| 130 | Chaetodon bennetti | DD | 91.33 | 81.62 | Wild-Caught |
| 131 | Chaetodon burgessi | LC | 598.32 | 105.80 | Wild-Caught |
| 132 | Chaetodon capistratus | LC | 52.49 | 46.04 | Wild-Caught |
| 133 | Chaetodon citrinellus | LC | 58.33 | 48.95 | Wild-Caught |
| 134 | Chaetodon collare | LC | 133.32 | 44.23 | Wild-Caught |
| 135 | Chaetodon daedalma | LC | 9999.99 | 3999.95 | Wild-Caught |
| 136 | Chaetodon declivis | LC | 749.99 | 236.22 | Wild-Caught |
| 137 | Chaetodon decussatus | LC | 53.32 | 53.28 | Wild-Caught |
| 138 | Chaetodon ephippium | LC | 83.33 | 76.24 | Wild-Caught |
| 139 | Chaetodon falcula | LC | 93.32 | 76.62 | Wild-Caught |
| 140 | Chaetodon fasciatus | LC | 293.66 | 114.95 | Wild-Caught |
| 141 | Chaetodon flavirostris | LC | 244.24 | 244.95 | Wild-Caught |
| 142 | Chaetodon fremblii | LC | 14.99 | 104.95 | Wild-Caught |
| 143 | Chaetodon guttatissimus | LC | 56.74 | 55.28 | Wild-Caught |
| 144 | Chaetodon humeralis | LC | 82.62 | 35.66 | Wild-Caught |
| 145 | Chaetodon kleinii | LC | 62.49 | 45.62 | Wild-Caught |
| 146 | Chaetodon larvatus | LC | 269.99 | 261.20 | Wild-Caught |
| 147 | Chaetodon lineolatus | LC | 97.99 | 56.95 | Wild-Caught |
| 148 | Chaetodon lunula | LC | 134.49 | 31.34 | Wild-Caught |
| 149 | Chaetodon melannotus | LC | 56.66 | 32.30 | Wild-Caught |
| 150 | Chaetodon mertensii | LC | 67.99 | 27.34 | Wild-Caught |
| 151 | Chaetodon meyeri | LC | 181.49 | 79.95 | Wild-Caught |
| 152 | Chaetodon miliaris | LC | 123.32 | 31.62 | Wild-Caught |
| 153 | Chaetodon mitratus | LC | 630.99 | 285.68 | Wild-Caught |
| 154 | Chaetodon ocellatus | LC | 49.99 | 75.99 | Wild-Caught |
| 155 | Chaetodon octofasciatus | LC | 104.99 | 31.62 | Wild-Caught |
| 156 | Chaetodon ornatissimus | LC | 89.99 | 63.28 | Wild-Caught |
| 157 | Chaetodon paucifasciatus | LC | 287.49 | 159.40 | Wild-Caught |
| 158 | Chaetodon pelewensis | LC | 60.99 | 46.48 | Wild-Caught |
| 159 | Chaetodon plebeius | LC | 83.32 | 53.28 | Wild-Caught |
| 160 | Chaetodon punctatofasciatus | LC | 72.49 | 36.62 | Wild-Caught |
| 161 | Chaetodon quadrimaculatus | LC | 69.99 | 69.95 | Wild-Caught |
| 162 | Chaetodon rafflesii | LC | 66.12 | 34.23 | Wild-Caught |
| 163 | Chaetodon rainfordi | NT | 262.62 | 101.20 | Wild-Caught |
| 164 | Chaetodon reticulatus | DD | 74.16 | 63.28 | Wild-Caught |
| 165 | Chaetodon semilarvatus | LC | 349.99 | 278.70 | Wild-Caught |
| 166 | Chaetodon speculum | LC | 61.66 | 63.28 | Wild-Caught |
| 167 | Chaetodon striatus | LC | 49.99 | 50.60 | Wild-Caught |
| 168 | Chaetodon tinkeri | LC | 974.49 | 699.95 | Wild-Caught |
| 169 | Chaetodon trichrous | LC | 172.49 | 74.57 | Wild-Caught |
| 170 | Chaetodon ulietensis | LC | 95.99 | 57.15 | Wild-Caught |
| 171 | Chaetodon unimaculatus | LC | 68.24 | 46.04 | Wild-Caught |
| 172 | Chaetodon vagabundus | LC | 60.41 | 36.23 | Wild-Caught |
| 173 | Chaetodon wiebeli | LC | 149.99 | 149.95 | Wild-Caught |
| 174 | Chaetodon xanthocephalus | LC | 81.66 | 70.59 | Wild-Caught |
| 175 | Chaetodon xanthurus | LC | 73.66 | 32.30 | Wild-Caught |
| 176 | Chaetodontoplus caeruleopunctatus | DD | 132.49 | 95.97 | Wild-Caught |
| 177 | Chaetodontoplus conspicillatus | LC | 9882.49 | 3499.95 | Wild-Caught |
| 178 | Chaetodontoplus duboulayi | LC | 427.49 | 295.47 | Wild-Caught, now Both |
| 179 | Chaetodontoplus melanosoma | LC | 99.99 | 91.23 | Wild-Caught |
| 180 | Chaetodontoplus meredithi | LC | 636.66 | 308.84 | Wild-Caught |
| 181 | Chaetodontoplus mesoleucus | LC | 62.32 | 24.37 | Wild-Caught |
| 182 | Chaetodontoplus septentrionalis | LC | 1095.32 | 368.28 | Wild-Caught |
| 183 | Cheilinus abudjubbe | LC | 209.99 | 139.95 | Wild-Caught |
| 184 | Cheilinus fasciatus | LC | 137.24 | 125.68 | Wild-Caught |
| 185 | Cheilinus lunulatus | LC | 673.48 | 337.45 | Wild-Caught |
| 186 | Cheilinus oxycephalus | LC | 49.99 | 24.95 | Wild-Caught |
| 187 | Cheilodipterus quinquelineatus | LC | 24.99 | 61.66 | Wild-Caught |
| 188 | Chelmon marginalis | LC | 692.74 | 157.45 | Wild-Caught |
| 189 | Chelmon muelleri | LC | 257.49 | 257.45 | Wild-Caught |
| 190 | Chelmon rostratus | LC | 176.49 | 47.89 | Wild-Caught |
| 191 | Choerodon fasciatus | LC | 305.08 | 154.25 | Wild-Caught |
| 192 | Choerodon jordani | LC | 148.32 | 76.62 | Wild-Caught |
| 193 | Chromis atripectoralis | LC | 19.99 | 74.86 | Wild-Caught |
| 194 | Chromis insolata | LC | 27.24 | 39.13 | Wild-Caught |
| 195 | Chromis limbaughi | LC | 48.74 | 36.46 | Wild-Caught |
| 196 | Chromis viridis | LC | 27.24 | 34.38 | Wild-Caught |
| 197 | Chromis weberi | LC | 18.99 | 34.99 | Wild-Caught |
| 198 | Chrysiptera cyanea | LC | 21.24 | 10.73 | Wild-Caught |
| 199 | Chrysiptera galba | LC | 149.99 | 49.95 | Wild-Caught |
| 200 | Chrysiptera hemicyanea | VU | 16.37 | 39.73 | Wild-Caught, now Both |
| 201 | Chrysiptera parasema | VU | 10.49 | 8.48 | Wild-Caught |
| 202 | Chrysiptera rollandi | LC | 14.99 | 14.95 | Wild-Caught |
| 203 | Chrysiptera springeri | LC | 14.99 | 12.64 | Wild-Caught |
| 204 | Chrysiptera starcki | LC | 100.49 | 29.95 | Wild-Caught |
| 205 | Chrysiptera talboti | LC | 14.49 | 46.50 | Wild-Caught |
| 206 | Chrysiptera taupou | LC | 21.24 | 39.99 | Wild-Caught |
| 207 | Cirrhilabrus adornatus | LC | 72.49 | 43.95 | Wild-Caught |
| 208 | Cirrhilabrus aurantidorsalis | LC | 119.99 | 64.97 | Wild-Caught |
| 209 | Cirrhilabrus balteatus | DD | 144.99 | 139.24 | Wild-Caught |
| 210 | Cirrhilabrus bathyphilus | LC | 199.99 | 70.48 | Wild-Caught |
| 211 | Cirrhilabrus condei | LC | 79.99 | 54.95 | Wild-Caught |
| 212 | Cirrhilabrus cyanopleura | DD | 79.43 | 48.12 | Wild-Caught |
| 213 | Cirrhilabrus exquisitus | DD | 105.44 | 117.97 | Wild-Caught |
| 214 | Cirrhilabrus filamentosus | DD | 62.49 | 43.31 | Wild-Caught |
| 215 | Cirrhilabrus flavidorsalis | LC | 82.49 | 49.60 | Wild-Caught |
| 216 | Cirrhilabrus jordani | LC | 552.87 | 101.48 | Wild-Caught |
| 217 | Cirrhilabrus laboutei | LC | 269.97 | 121.47 | Wild-Caught |
| 218 | Cirrhilabrus lineatus | LC | 237.77 | 152.85 | Wild-Caught |
| 219 | Cirrhilabrus lubbocki | LC | 52.49 | 79.30 | Wild-Caught |
| 220 | Cirrhilabrus lunatus | LC | 355.06 | 524.95 | Wild-Caught |
| 221 | Cirrhilabrus luteovittatus | LC | 172.29 | 100.50 | Wild-Caught |
| 222 | Cirrhilabrus naokoae | NE | 122.49 | 229.95 | Wild-Caught |
| 223 | Cirrhilabrus punctatus | LC | 58.12 | 26.82 | Wild-Caught |
| 224 | Cirrhilabrus pylei | LC | 142.74 | 169.95 | Wild-Caught |
| 225 | Cirrhilabrus rhomboidalis | DD | 324.99 | 293.81 | Wild-Caught |
| 226 | Cirrhilabrus roseafascia | LC | 409.99 | 126.62 | Wild-Caught |
| 227 | Cirrhilabrus rubrimarginatus | LC | 118.74 | 46.46 | Wild-Caught |
| 228 | Cirrhilabrus rubripinnis | LC | 64.99 | 44.95 | Wild-Caught |
| 229 | Cirrhilabrus rubrisquamis | DD | 110.99 | 91.98 | Wild-Caught |
| 230 | Cirrhilabrus rubriventralis | LC | 49.99 | 32.98 | Wild-Caught |
| 231 | Cirrhilabrus scottorum | LC | 202.49 | 93.94 | Wild-Caught |
| 232 | Cirrhilabrus solorensis | DD | 86.99 | 51.80 | Wild-Caught |
| 233 | Cirrhilabrus temminckii | DD | 79.99 | 64.95 | Wild-Caught |
| 234 | Cirrhilabrus tonozukai | DD | 69.99 | 67.45 | Wild-Caught |
| 235 | Cirripectes stigmaticus | LC | 66.99 | 144.65 | Wild-Caught |
| 236 | Compsanthias ventralis | LC | 344.69 | 149.95 | Wild-Caught |
| 237 | Coradion altivelis | LC | 311.91 | 203.28 | Wild-Caught |
| 238 | Coris aygula | VU | 72.99 | 56.95 | Wild-Caught |
| 239 | Coris formosa | LC | 96.78 | 92.43 | Wild-Caught |
| 240 | Coris gaimard | LC | 87.78 | 55.70 | Wild-Caught |
| 241 | Coryphopterus personatus | VU | 22.74 | 21.95 | Wild-Caught |
| 242 | Cromileptes altivelis | DD | 53.74 | 64.34 | Wild-Caught |
| 243 | Crossosalarias macrospilus | LC | 29.99 | 22.47 | Wild-Caught |
| 244 | Cryptocentrus cinctus | LC | 17.69 | 16.96 | Wild-Caught, now Both |
| 245 | Cryptocentrus leptocephalus | LC | 37.66 | 21.58 | Wild-Caught |
| 246 | Cryptocentrus pavoninoides | DD | 71.24 | 31.33 | Wild-Caught |
| 247 | Ctenochaetus binotatus | LC | 71.66 | 33.18 | Wild-Caught |
| 248 | Ctenochaetus flavicauda | LC | 332.49 | 17.32 | Wild-Caught |
| 249 | Ctenochaetus hawaiiensis | LC | 474.99 | 139.88 | Wild-Caught |
| 250 | Ctenochaetus striatus | LC | 84.62 | 39.36 | Wild-Caught |
| 251 | Ctenochaetus strigosus | LC | 181.24 | 51.11 | Wild-Caught |
| 252 | Ctenochaetus tominiensis | LC | 83.33 | 106.65 | Wild-Caught |
| 253 | Ctenochaetus truncatus | LC | 106.66 | 85.47 | Wild-Caught |
| 254 | Ctenogobiops tangaroai | LC | 39.99 | 23.97 | Wild-Caught |
| 255 | Cypho purpurascens | LC | 199.99 | 129.95 | Wild-Caught |
| 256 | Dactylopus dactylopus | LC | 41.66 | 42.28 | Wild-Caught |
| 257 | Dascyllus albisella | LC | 37.49 | 19.45 | Wild-Caught |
| 258 | Dascyllus aruanus | LC | 9.99 | 15.63 | Wild-Caught |
| 259 | Dascyllus auripinnis | LC | 39.99 | 18.45 | Wild-Caught |
| 260 | Dascyllus melanurus | LC | 12.49 | 5.47 | Wild-Caught |
| 261 | Dascyllus reticulatus | LC | 9.99 | 18.64 | Wild-Caught |
| 262 | Dascyllus trimaculatus | VU | 10.99 | 49.73 | Wild-Caught |
| 263 | Discordipinna griessingeri | LC | 132.66 | 99.95 | Wild-Caught |
| 264 | Ecsenius bicolor | LC | 36.74 | 42.42 | Wild-Caught |
| 265 | Ecsenius bimaculatus | LC | 23.66 | 34.14 | Wild-Caught |
| 266 | Ecsenius gravieri | LC | 249.99 | 124.95 | Wild-Caught |
| 267 | Ecsenius lineatus | LC | 43.49 | 23.73 | Wild-Caught |
| 268 | Ecsenius lividanalis | LC | 54.99 | 19.95 | Wild-Caught |
| 269 | Ecsenius melarchus | LC | 24.99 | 24.95 | Wild-Caught |
| 270 | Ecsenius midas | LC | 77.99 | 50.72 | Wild-Caught |
| 271 | Ecsenius namiyei | LC | 29.99 | 47.84 | Wild-Caught |
| 272 | Ecsenius stigmatura | LC | 29.99 | 55.79 | Wild-Caught |
| 273 | Elacatinus evelynae | LC | 24.49 | 32.47 | Captive-Bred, now Both |
| 274 | Elacatinus figaro | VU | 20.74 | 23.99 | Captive-Bred |
| 275 | Elacatinus horsti | LC | 49.99 | 49.95 | Captive-Bred, now Wild-Caught |
| 276 | Elacatinus oceanops | LC | 39.99 | 23.97 | Captive-Bred, now Both |
| 277 | Elacatinus puncticulatus | LC | 37.99 | 24.95 | Wild-Caught, now Both |
| 278 | Emblemaria pandionis | LC | 32.49 | 34.95 | Wild-Caught |
| 279 | Epibulus insidiator | LC | 83.32 | 66.62 | Wild-Caught |
| 280 | Epinephelus flavocaeruleus | LC | 397.34 | 231.95 | Wild-Caught |
| 281 | Epinephelus lanceolatus | DD | 874.99 | 874.95 | Wild-Caught |
| 282 | Eviota pellucida | LC | 24.99 | 29.99 | Wild-Caught |
| 283 | Exallias brevis | LC | 94.99 | 64.95 | Wild-Caught |
| 284 | Forcipiger flavissimus | LC | 94.87 | 40.81 | Wild-Caught |
| 285 | Fowleria flammea | LC | 33.49 | 29.99 | Wild-Caught |
| 286 | Fowleria isostigma | NE | 22.99 | 119.99 | Wild-Caught |
| 287 | Genicanthus bellus | LC | 410.79 | 128.50 | Wild-Caught |
| 288 | Genicanthus caudovittatus | LC | 280.79 | 179.95 | Wild-Caught |
| 289 | Genicanthus lamarck | LC | 93.49 | 76.60 | Wild-Caught |
| 290 | Genicanthus melanospilos | LC | 99.99 | 56.00 | Wild-Caught |
| 291 | Genicanthus semifasciatus | LC | 352.91 | 274.95 | Wild-Caught |
| 292 | Genicanthus watanabei | LC | 389.90 | 84.77 | Wild-Caught |
| 293 | Gobiodon albofasciatus | LC | 29.99 | 9.95 | Wild-Caught |
| 294 | Gobiodon atrangulatus | DD | 17.49 | 19.24 | Wild-Caught |
| 295 | Gobiodon ceramensis | NT | 19.99 | 9.95 | Wild-Caught |
| 296 | Gobiodon citrinus | EN | 32.70 | 58.40 | Wild-Caught |
| 297 | Gobiodon histrio | LC | 18.49 | 9.47 | Wild-Caught |
| 298 | Gobiodon okinawae | LC | 17.49 | 22.58 | Wild-Caught |
| 299 | Gobiodon quinquestrigatus | LC | 19.99 | 7.95 | Wild-Caught |
| 300 | Gomphosus varius | LC | 102.71 | 41.78 | Wild-Caught |
| 301 | Gunnellichthys curiosus | LC | 47.49 | 42.45 | Wild-Caught |
| 302 | Halichoeres biocellatus | LC | 57.49 | 46.49 | Wild-Caught |
| 303 | Halichoeres bivittatus | LC | 49.98 | 24.99 | Wild-Caught |
| 304 | Halichoeres chloropterus | LC | 39.32 | 29.41 | Wild-Caught |
| 305 | Halichoeres chrysotaenia | LC | 54.37 | 45.68 | Wild-Caught |
| 306 | Halichoeres chrysus | LC | 44.99 | 47.22 | Wild-Caught, now Both |
| 307 | Halichoeres cosmetus | LC | 58.74 | 116.32 | Wild-Caught |
| 308 | Halichoeres garnoti | LC | 49.49 | 58.64 | Wild-Caught |
| 309 | Halichoeres hortulanus | LC | 55.87 | 44.86 | Wild-Caught |
| 310 | Halichoeres iridis | LC | 96.66 | 71.06 | Wild-Caught |
| 311 | Halichoeres leucoxanthus | LC | 48.87 | 53.24 | Wild-Caught |
| 312 | Halichoeres leucurus | LC | 59.37 | 78.47 | Wild-Caught |
| 313 | Halichoeres marginatus | VU | 59.99 | 39.95 | Wild-Caught |
| 314 | Halichoeres melanurus | LC | 58.24 | 36.05 | Wild-Caught |
| 315 | Halichoeres miniatus | LC | 39.99 | 40.32 | Wild-Caught |
| 316 | Halichoeres ornatissimus | LC | 89.99 | 51.92 | Wild-Caught |
| 317 | Halichoeres prosopeion | LC | 29.99 | 50.32 | Wild-Caught |
| 318 | Halichoeres radiatus | LC | 69.16 | 74.95 | Wild-Caught |
| 319 | Halichoeres richmondi | LC | 83.32 | 94.41 | Wild-Caught |
| 320 | Halichoeres rubricephalus | DD | 159.99 | 159.95 | Wild-Caught |
| 321 | Hemigymnus fasciatus | LC | 43.32 | 43.28 | Wild-Caught |
| 322 | Hemigymnus melapterus | LC | 34.99 | 28.28 | Wild-Caught |
| 323 | Hemitaurichthys polylepis | LC | 318.24 | 59.95 | Wild-Caught |
| 324 | Hemitaurichthys zoster | LC | 123.32 | 89.95 | Wild-Caught |
| 325 | Heniochus acuminatus | LC | 92.49 | 39.51 | Wild-Caught |
| 326 | Heniochus diphreutes | LC | 120.99 | 84.99 | Wild-Caught |
| 327 | Heniochus intermedius | LC | 373.99 | 101.14 | Wild-Caught |
| 328 | Heniochus varius | LC | 76.99 | 37.22 | Wild-Caught |
| 329 | Holacanthus africanus | LC | 557.54 | 459.95 | Wild-Caught |
| 330 | Holacanthus bermudensis | LC | 157.41 | 138.34 | Wild-Caught |
| 331 | Holacanthus ciliaris | LC | 289.71 | 139.92 | Wild-Caught |
| 332 | Holacanthus passer | LC | 395.29 | 237.08 | Wild-Caught |
| 333 | Holacanthus tricolor | LC | 91.82 | 46.80 | Wild-Caught |
| 334 | Hypoplectrus indigo | LC | 53.32 | 46.62 | Wild-Caught |
| 335 | Johnrandallia nigrirostris | LC | 39.49 | 78.28 | Wild-Caught |
| 336 | Koumansetta hectori | LC | 29.99 | 18.31 | Wild-Caught |
| 337 | Koumansetta rainfordi | LC | 34.99 | 46.35 | Wild-Caught, now Both |
| 338 | Labracinus cyclophthalmus | LC | 77.74 | 33.12 | Wild-Caught |
| 339 | Labroides bicolor | LC | 89.99 | 39.95 | Wild-Caught |
| 340 | Labroides dimidiatus | LC | 32.74 | 73.45 | Wild-Caught |
| 341 | Labroides pectoralis | LC | 99.99 | 29.95 | Wild-Caught |
| 342 | Labroides phthirophagus | LC | 129.99 | 59.95 | Wild-Caught |
| 343 | Labropsis alleni | LC | 28.99 | 29.95 | Wild-Caught |
| 344 | Larabicus quadrilineatus | DD | 149.99 | 44.99 | Wild-Caught |
| 345 | Liopropoma carmabi | LC | 1414.99 | 999.95 | Wild-Caught |
| 346 | Liopropoma mitratum | LC | 166.66 | 29.99 | Wild-Caught |
| 347 | Liopropoma rubre | LC | 264.99 | 149.95 | Wild-Caught |
| 348 | Liopropoma susumi | LC | 134.99 | 109.95 | Wild-Caught |
| 349 | Liopropoma swalesi | LC | 129.99 | 74.32 | Wild-Caught |
| 350 | Lythrypnus dalli | LC | 46.66 | 31.98 | Wild-Caught |
| 351 | Lythrypnus zebra | LC | 59.99 | 23.97 | Wild-Caught |
| 352 | Macropharyngodon bipartitus | LC | 103.99 | 55.14 | Wild-Caught |
| 353 | Macropharyngodon choati | LC | 299.99 | 249.95 | Wild-Caught |
| 354 | Macropharyngodon geoffroy | LC | 133.99 | 83.28 | Wild-Caught |
| 355 | Macropharyngodon kuiteri | LC | 287.36 | 194.95 | Wild-Caught |
| 356 | Macropharyngodon meleagris | LC | 89.16 | 49.30 | Wild-Caught |
| 357 | Macropharyngodon negrosensis | LC | 71.38 | 34.97 | Wild-Caught |
| 358 | Macropharyngodon ornatus | LC | 69.49 | 49.95 | Wild-Caught |
| 359 | Manonichthys alleni | LC | 81.66 | 59.99 | Wild-Caught |
| 360 | Manonichthys polynemus | DD | 44.99 | 44.95 | Wild-Caught |
| 361 | Manonichthys splendens | LC | 54.99 | 56.23 | Wild-Caught, now Both |
| 362 | Meiacanthus atrodorsalis | LC | 26.55 | 25.27 | Wild-Caught |
| 363 | Meiacanthus bundoon | LC | 57.49 | 39.95 | Wild-Caught, now Both |
| 364 | Meiacanthus grammistes | LC | 39.99 | 150.48 | Wild-Caught, now Both |
| 365 | Meiacanthus mossambicus | LC | 37.49 | 31.63 | Wild-Caught, now Both |
| 366 | Meiacanthus nigrolineatus | LC | 47.99 | 29.97 | Captive-Bred |
| 367 | Meiacanthus oualanensis | LC | 34.99 | 109.64 | Wild-Caught, now Both |
| 368 | Meiacanthus smithi | LC | 36.99 | 25.30 | Wild-Caught, now Both |
| 369 | Meiacanthus tongaensis | LC | 33.99 | 77.48 | Wild-Caught |
| 370 | Microspathodon chrysurus | LC | 29.99 | 19.95 | Wild-Caught |
| 371 | Naso brevirostris | LC | 111.99 | 106.64 | Wild-Caught |
| 372 | Naso elegans | LC | 203.56 | 115.43 | Wild-Caught |
| 373 | Naso lituratus | LC | 227.97 | 90.80 | Wild-Caught |
| 374 | Naso lopezi | LC | 83.33 | 42.21 | Wild-Caught |
| 375 | Naso unicornis | LC | 202.85 | 146.85 | Wild-Caught |
| 376 | Naso vlamingii | LC | 297.61 | 182.07 | Wild-Caught |
| 377 | Nemanthias bartlettorum | LC | 92.49 | 85.28 | Wild-Caught |
| 378 | Nemanthias bicolor | LC | 91.66 | 105.61 | Wild-Caught |
| 379 | Nemanthias carberryi | LC | 74.99 | 49.99 | Wild-Caught |
| 380 | Nemanthias dispar | LC | 57.49 | 25.98 | Wild-Caught |
| 381 | Nemateleotris decora | LC | 60.87 | 34.10 | Wild-Caught |
| 382 | Nemateleotris helfrichi | LC | 149.99 | 89.98 | Wild-Caught |
| 383 | Nemateleotris magnifica | LC | 25.27 | 20.23 | Wild-Caught |
| 384 | Neoglyphidodon crossi | LC | 14.99 | 9.99 | Wild-Caught |
| 385 | Neoglyphidodon melas | LC | 11.24 | 7.76 | Wild-Caught |
| 386 | Neoglyphidodon nigroris | LC | 14.49 | 7.95 | Wild-Caught |
| 387 | Neoglyphidodon oxyodon | LC | 19.99 | 13.23 | Wild-Caught |
| 388 | Neoglyphidodon polyacanthus | LC | 9.99 | 9.95 | Wild-Caught |
| 389 | Neopomacentrus bankieri | LC | 19.99 | 5.95 | Wild-Caught |
| 390 | Neopomacentrus cyanomos | LC | 12.99 | 53.32 | Wild-Caught, now Both |
| 391 | Neosynchiropus ocellatus | LC | 24.14 | 51.85 | Wild-Caught |
| 392 | Novaculichthys taeniourus | LC | 71.78 | 49.57 | Wild-Caught |
| 393 | Novaculoides macrolepidotus | LC | 134.99 | 54.98 | Wild-Caught |
| 394 | Odontanthias borbonius | LC | 999.99 | 249.95 | Wild-Caught |
| 395 | Ogilbyina novaehollandiae | LC | 199.99 | 149.95 | Wild-Caught |
| 396 | Ophioblennius atlanticus | LC | 38.99 | 26.68 | Wild-Caught |
| 397 | Ostorhinchus cyanosoma | LC | 29.99 | 32.48 | Wild-Caught |
| 398 | Ostorhinchus parvulus | LC | 26.99 | 24.95 | Wild-Caught |
| 399 | Oxycheilinus bimaculatus | LC | 59.99 | 72.10 | Wild-Caught |
| 400 | Oxymonacanthus longirostris | VU | 58.74 | 44.95 | Wild-Caught |
| 401 | Paracanthurus hepatus | LC | 153.87 | 70.26 | Wild-Caught, now Both |
| 402 | Paracentropyge multifasciata | LC | 260.59 | 88.70 | Wild-Caught |
| 403 | Paracheilinus angulatus | LC | 69.99 | 79.95 | Wild-Caught |
| 404 | Paracheilinus carpenteri | DD | 52.99 | 38.38 | Wild-Caught |
| 405 | Paracheilinus cyaneus | LC | 76.49 | 38.23 | Wild-Caught |
| 406 | Paracheilinus filamentosus | LC | 69.99 | 36.97 | Wild-Caught |
| 407 | Paracheilinus flavianalis | LC | 64.99 | 37.97 | Wild-Caught |
| 408 | Paracheilinus lineopunctatus | LC | 75.99 | 52.98 | Wild-Caught |
| 409 | Paracheilinus mccoskeri | LC | 79.99 | 53.91 | Wild-Caught |
| 410 | Paracheilinus octotaenia | LC | 147.66 | 189.95 | Wild-Caught |
| 411 | Paracheilinus rubricaudalis | LC | 179.99 | 52.48 | Wild-Caught |
| 412 | Paragobiodon lacunicolus | LC | 30.49 | 35.51 | Wild-Caught |
| 413 | Paraluteres prionurus | LC | 46.87 | 48.70 | Wild-Caught |
| 414 | Pervagor melanocephalus | LC | 52.49 | 73.73 | Wild-Caught |
| 415 | Pervagor spilosoma | LC | 64.99 | 28.28 | Wild-Caught |
| 416 | Pholidichthys leucotaenia | NE | 18.99 | 24.37 | Wild-Caught |
| 417 | Pictichromis diadema | LC | 22.49 | 32.98 | Wild-Caught |
| 418 | Pictichromis paccagnellorum | LC | 23.49 | 21.13 | Wild-Caught |
| 419 | Pictichromis porphyrea | LC | 23.49 | 46.65 | Wild-Caught |
| 420 | Plectorhinchus albovittatus | NE | 42.32 | 38.95 | Wild-Caught |
| 421 | Plectorhinchus chaetodonoides | NE | 59.99 | 42.40 | Wild-Caught |
| 422 | Plectorhinchus diagrammus | NE | 49.99 | 25.28 | Wild-Caught |
| 423 | Plectorhinchus picus | NE | 59.99 | 49.95 | Wild-Caught |
| 424 | Plectorhinchus vittatus | LC | 52.32 | 31.28 | Wild-Caught |
| 425 | Plectranthias inermis | LC | 59.99 | 49.95 | Wild-Caught |
| 426 | Pogonoperca punctata | LC | 129.99 | 114.24 | Wild-Caught |
| 427 | Pomacanthus annularis | LC | 305.30 | 101.58 | Wild-Caught |
| 428 | Pomacanthus arcuatus | LC | 151.55 | 112.90 | Wild-Caught |
| 429 | Pomacanthus asfur | LC | 334.99 | 189.34 | Wild-Caught |
| 430 | Pomacanthus chrysurus | LC | 601.42 | 215.45 | Wild-Caught |
| 431 | Pomacanthus imperator | LC | 670.93 | 141.88 | Wild-Caught |
| 432 | Pomacanthus maculosus | LC | 356.66 | 226.08 | Wild-Caught |
| 433 | Pomacanthus navarchus | LC | 246.66 | 133.35 | Wild-Caught, now Both |
| 434 | Pomacanthus paru | LC | 270.30 | 144.71 | Wild-Caught |
| 435 | Pomacanthus semicirculatus | LC | 136.21 | 78.07 | Wild-Caught |
| 436 | Pomacanthus sexstriatus | LC | 162.49 | 63.22 | Wild-Caught |
| 437 | Pomacanthus xanthometopon | LC | 232.49 | 98.14 | Wild-Caught |
| 438 | Pomacanthus zonipectus | LC | 235.35 | 192.26 | Wild-Caught |
| 439 | Pomacentrus alleni | LC | 25.49 | 10.64 | Wild-Caught |
| 440 | Pomacentrus auriventris | LC | 14.99 | 8.26 | Wild-Caught |
| 441 | Pomacentrus bankanensis | LC | 14.99 | 12.95 | Wild-Caught |
| 442 | Pomacentrus coelestis | LC | 16.99 | 14.63 | Wild-Caught |
| 443 | Pomacentrus moluccensis | LC | 14.99 | 7.47 | Wild-Caught, now Both |
| 444 | Pomacentrus similis | LC | 4.99 | 79.99 | Wild-Caught |
| 445 | Pomacentrus smithi | LC | 6.99 | 9.99 | Wild-Caught, now Both |
| 446 | Pomacentrus vaiuli | LC | 72.99 | 34.95 | Wild-Caught |
| 447 | Priolepis nocturna | LC | 74.99 | 39.97 | Wild-Caught |
| 448 | Prognathodes aculeatus | LC | 142.37 | 133.28 | Wild-Caught |
| 449 | Pseudanthias bimaculatus | LC | 130.99 | 52.77 | Wild-Caught |
| 450 | Pseudanthias evansi | LC | 72.49 | 44.50 | Wild-Caught |
| 451 | Pseudanthias fasciatus | LC | 98.74 | 98.70 | Wild-Caught |
| 452 | Pseudanthias huchtii | LC | 56.66 | 39.95 | Wild-Caught |
| 453 | Pseudanthias hypselosoma | LC | 45.04 | 61.59 | Wild-Caught |
| 454 | Pseudanthias ignitus | LC | 48.37 | 36.10 | Wild-Caught |
| 455 | Pseudanthias luzonensis | LC | 96.49 | 53.09 | Wild-Caught |
| 456 | Pseudanthias pictilis | LC | 219.56 | 56.49 | Wild-Caught |
| 457 | Pseudanthias pleurotaenia | LC | 98.66 | 150.74 | Wild-Caught |
| 458 | Pseudanthias pulcherrimus | LC | 65.49 | 50.81 | Wild-Caught |
| 459 | Pseudanthias randalli | LC | 67.66 | 45.30 | Wild-Caught |
| 460 | Pseudanthias rubrizonatus | LC | 62.66 | 46.77 | Wild-Caught |
| 461 | Pseudanthias squamipinnis | LC | 64.31 | 36.87 | Wild-Caught |
| 462 | Pseudanthias tuka | LC | 64.33 | 36.62 | Wild-Caught |
| 463 | Pseudocheilinops ataenia | LC | 69.99 | 48.31 | Wild-Caught |
| 464 | Pseudocheilinus evanidus | LC | 44.99 | 50.98 | Wild-Caught |
| 465 | Pseudocheilinus hexataenia | LC | 31.99 | 17.23 | Wild-Caught |
| 466 | Pseudocheilinus ocellatus | LC | 179.99 | 79.98 | Wild-Caught |
| 467 | Pseudocheilinus octotaenia | LC | 44.99 | 39.47 | Wild-Caught |
| 468 | Pseudocheilinus tetrataenia | LC | 150.49 | 69.95 | Wild-Caught |
| 469 | Pseudochromis aldabraensis | LC | 39.99 | 57.22 | Captive-Bred |
| 470 | Pseudochromis coccinicauda | LC | 39.49 | 48.73 | Wild-Caught |
| 471 | Pseudochromis cyanotaenia | LC | 29.99 | 29.95 | Wild-Caught |
| 472 | Pseudochromis dilectus | LC | 50.82 | 39.15 | Wild-Caught |
| 473 | Pseudochromis elongatus | LC | 49.99 | 57.47 | Wild-Caught, now Both |
| 474 | Pseudochromis flavivertex | LC | 53.49 | 49.47 | Captive-Bred, now Both |
| 475 | Pseudochromis fridmani | LC | 57.49 | 99.37 | Wild-Caught, now Both |
| 476 | Pseudochromis fuscus | LC | 44.99 | 26.47 | Wild-Caught |
| 477 | Pseudochromis sankeyi | LC | 72.99 | 39.49 | Captive-Bred |
| 478 | Pseudochromis springeri | LC | 59.99 | 50.98 | Captive-Bred |
| 479 | Pseudochromis steenei | LC | 69.99 | 69.95 | Wild-Caught |
| 480 | Pseudochromis tonozukai | LC | 74.99 | 72.71 | Wild-Caught |
| 481 | Pseudodax moluccanus | LC | 69.16 | 44.28 | Wild-Caught |
| 482 | Pseudojuloides cerasinus | DD | 44.99 | 37.45 | Wild-Caught |
| 483 | Pseudojuloides severnsi | LC | 75.43 | 47.46 | Wild-Caught |
| 484 | Pteragogus cryptus | LC | 39.90 | 39.95 | Wild-Caught |
| 485 | Pterapogon kauderni | EN | 39.99 | 34.86 | Captive-Bred, now Both |
| 486 | Ptereleotris evides | LC | 32.99 | 37.58 | Wild-Caught |
| 487 | Ptereleotris hanae | LC | 53.74 | 54.18 | Wild-Caught |
| 488 | Ptereleotris heteroptera | LC | 37.49 | 28.22 | Wild-Caught |
| 489 | Ptereleotris microlepis | LC | 33.74 | 21.45 | Wild-Caught |
| 490 | Ptereleotris zebra | LC | 44.68 | 29.15 | Wild-Caught |
| 491 | Pycnochromis agilis | LC | 29.99 | 51.66 | Wild-Caught |
| 492 | Pycnochromis amboinensis | LC | 17.99 | 17.95 | Wild-Caught |
| 493 | Pycnochromis margaritifer | LC | 24.66 | 48.73 | Wild-Caught |
| 494 | Pycnochromis retrofasciatus | LC | 16.49 | 8.95 | Wild-Caught |
| 495 | Pycnochromis vanderbilti | LC | 37.49 | 24.95 | Wild-Caught |
| 496 | Pygoplites diacanthus | LC | 301.35 | 131.86 | Wild-Caught |
| 497 | Pyronotanthias flavoguttatus | LC | 103.49 | 74.45 | Wild-Caught |
| 498 | Pyronotanthias lori | LC | 61.66 | 62.21 | Wild-Caught |
| 499 | Pyronotanthias parvirostris | LC | 73.74 | 157.86 | Wild-Caught |
| 500 | Pyronotanthias smithvanizi | LC | 129.99 | 49.95 | Wild-Caught |
| 501 | Salarias fasciatus | LC | 33.74 | 41.55 | Wild-Caught |
| 502 | Salarias ramosus | LC | 37.24 | 42.59 | Wild-Caught |
| 503 | Salarias segmentatus | LC | 39.99 | 89.97 | Wild-Caught |
| 504 | Scartella cristata | LC | 44.42 | 79.99 | Wild-Caught, now Both |
| 505 | Scarus taeniopterus | LC | 59.32 | 59.28 | Wild-Caught |
| 506 | Serranocirrhitus latus | LC | 144.99 | 66.10 | Wild-Caught |
| 507 | Serranus baldwini | LC | 24.99 | 46.23 | Wild-Caught |
| 508 | Serranus tabacarius | LC | 48.53 | 35.12 | Wild-Caught |
| 509 | Serranus tigrinus | LC | 30.57 | 35.63 | Wild-Caught |
| 510 | Serranus tortugarum | LC | 46.99 | 38.30 | Wild-Caught, now Both |
| 511 | Signigobius biocellatus | LC | 30.66 | 18.97 | Wild-Caught |
| 512 | Sphaeramia nematoptera | LC | 28.99 | 29.76 | Wild-Caught, now Both |
| 513 | Sphaeramia orbicularis | LC | 19.99 | 17.14 | Wild-Caught |
| 514 | Stegastes lacrymatus | LC | 10.49 | 11.97 | Wild-Caught |
| 515 | Stethojulis balteata | LC | 44.99 | 44.95 | Wild-Caught |
| 516 | Stethojulis bandanensis | LC | 84.99 | 41.45 | Wild-Caught |
| 517 | Stonogobiops dracula | LC | 177.99 | 92.47 | Wild-Caught |
| 518 | Stonogobiops nematodes | LC | 34.99 | 39.56 | Wild-Caught |
| 519 | Stonogobiops yasha | LC | 89.99 | 52.58 | Wild-Caught, now Both |
| 520 | Synchiropus picturatus | LC | 39.99 | 26.19 | Wild-Caught, now Both |
| 521 | Synchiropus splendidus | LC | 62.00 | 36.39 | Wild-Caught |
| 522 | Synchiropus stellatus | LC | 38.74 | 28.88 | Wild-Caught, now Both |
| 523 | Thalassoma amblycephalum | LC | 44.99 | 18.99 | Wild-Caught |
| 524 | Thalassoma bifasciatum | LC | 37.49 | 27.91 | Wild-Caught |
| 525 | Thalassoma duperrey | LC | 96.66 | 67.18 | Wild-Caught |
| 526 | Thalassoma hardwicke | LC | 69.99 | 59.87 | Wild-Caught |
| 527 | Thalassoma hebraicum | LC | 71.24 | 60.25 | Wild-Caught |
| 528 | Thalassoma jansenii | LC | 56.99 | 114.62 | Wild-Caught |
| 529 | Thalassoma lucasanum | LC | 79.99 | 59.85 | Wild-Caught |
| 530 | Thalassoma lunare | LC | 71.05 | 128.45 | Wild-Caught |
| 531 | Thalassoma lutescens | LC | 138.63 | 79.75 | Wild-Caught |
| 532 | Thalassoma purpureum | LC | 155.99 | 80.95 | Wild-Caught |
| 533 | Thalassoma quinquevittatum | LC | 89.85 | 70.28 | Wild-Caught |
| 534 | Thalassoma rueppellii | LC | 249.99 | 169.97 | Wild-Caught |
| 535 | Tigrigobius macrodon | LC | 33.49 | 21.47 | Captive-Bred |
| 536 | Tigrigobius multifasciatus | LC | 29.99 | 65.31 | Wild-Caught, now Both |
| 537 | Tomiyamichthys oni | LC | 27.49 | 24.95 | Wild-Caught |
| 538 | Trichonotus setiger | LC | 46.66 | 46.62 | Wild-Caught |
| 539 | Trimma cana | LC | 42.49 | 29.95 | Wild-Caught |
| 540 | Valenciennea helsdingenii | LC | 52.49 | 34.95 | Wild-Caught |
| 541 | Valenciennea longipinnis | LC | 34.99 | 28.58 | Wild-Caught, now Both |
| 542 | Valenciennea puellaris | LC | 46.95 | 36.56 | Wild-Caught |
| 543 | Valenciennea sexguttata | LC | 41.80 | 23.91 | Wild-Caught |
| 544 | Valenciennea strigata | LC | 53.99 | 24.77 | Wild-Caught |
| 545 | Valenciennea wardii | LC | 58.32 | 33.93 | Wild-Caught |
| 546 | Variola louti | LC | 102.24 | 43.08 | Wild-Caught |
| 547 | Wetmorella albofasciata | LC | 70.49 | 154.97 | Wild-Caught |
| 548 | Wetmorella nigropinnata | LC | 89.74 | 38.31 | Wild-Caught |
| 549 | Wetmorella tanakai | DD | 64.99 | 154.97 | Wild-Caught |
| 550 | Zebrasoma desjardinii | LC | 168.33 | 91.57 | Wild-Caught |
| 551 | Zebrasoma flavescens | LC | 270.35 | 46.07 | Wild-Caught, now Both |
| 552 | Zebrasoma rostratum | DD | 2090.99 | 1179.95 | Wild-Caught |
| 553 | Zebrasoma scopas | LC | 80.32 | 43.42 | Wild-Caught |
| 554 | Zebrasoma velifer | LC | 93.12 | 63.37 | Wild-Caught |
| 555 | Zebrasoma xanthurum | VU | 399.99 | 223.13 | Wild-Caught |
| 556 | Zoramia leptacanthus | LC | 29.99 | 28.31 | Wild-Caught |

# References

Dee, L., Karr, K., Landesberg, C. & Thornhill, D. (2019). Assessing Vulnerability of Fish in the U.S. Marine Aquarium Trade. *Front. Mar. Sci.*, 5, 527.

Holmberg, R.J., Tlusty, M.F., Futoma, E., Kaufman, L., Morris, J.A. & Rhyne, A.L. (2015). The 800-Pound Grouper in the Room: Asymptotic Body Size and Invasiveness of Marine Aquarium Fishes. *Marine Policy*, 53, 7–12.

Kay, S. & Hoyle, S. (2001). Mail Order, the Internet, and Invasive Aquatic Weeds. *Journal of Aquatic Plant Management*.

Rhyne, A., Tlusty, M., Schofield, P., Kaufman, L., Morris, J. & Bruckner, A. (2012). Revealing the Appetite of the Marine Aquarium Fish Trade: The Volume and Biodiversity of Fish Imported into the United States. *PLOS ONE*, 7, e35808.

Rhyne, A.L., Tlusty, M.F., Szczebak, J.T. & Holmberg, R.J. (2017). Expanding our understanding of the trade in marine aquarium animals. *PeerJ*, 5, e2949.

Sinha, A., Pandey, P.K. & Ghosh, S. (2023). Editorial: Ornamental fishing industry. *Frontiers in Marine Science*, 10.

Smith, K., Behrens, M., Max, L. & Daszak, P. (2008). U.S. drowning in unidentified fishes: Scope, implications, and regulation of live fish import. *Conservation Letters*, 1, 103–109.

Stringham, O.C., Toomes, A., Kanishka, A.M., Mitchell, L., Heinrich, S., Ross, J.V. & Cassey, P. (2021). A guide to using the internet to monitor and quantify the wildlife trade. *Conservation Biology*, 35, 1130–1139.

Tissot, B.N., Best, B.A., Borneman, E.H., Bruckner, A.W., Cooper, C.H., D’Agnes, H., Fitzgerald, T.P., Leland, A., Lieberman, S., Mathews Amos, A., Sumaila, R., Telecky, T.M., McGilvray, F., Plankis, B.J., Rhyne, A.L., Roberts, G.G., Starkhouse, B. & Stevenson, T.C. (2010). How U.S. ocean policy and market power can reform the coral reef wildlife trade. *Marine Policy*, 34, 1385–1388.

Wabnitz, C. (2003). *From ocean to aquarium : the global trade in marine ornamental species*. Cambridge : UNEP World Conservation Monitoring Centre.
